# Stability and mechanism of threose nucleic acid toward acid-mediated degradation

**DOI:** 10.1093/nar/gkad716

**Published:** 2023-08-31

**Authors:** Erica M Lee, Noah A Setterholm, Mohammad Hajjar, Bhawna Barpuzary, John C Chaput

**Affiliations:** Department of Pharmaceutical Sciences, University of California, Irvine, CA 92697-3958, USA; Department of Pharmaceutical Sciences, University of California, Irvine, CA 92697-3958, USA; Department of Pharmaceutical Sciences, University of California, Irvine, CA 92697-3958, USA; Department of Pharmaceutical Sciences, University of California, Irvine, CA 92697-3958, USA; Department of Pharmaceutical Sciences, University of California, Irvine, CA 92697-3958, USA; Department of Chemistry, University of California, Irvine, CA 92697-3958, USA; Department of Molecular Biology and Biochemistry, University of California, Irvine, CA 92697-3958, USA; Department of Chemical and Biomolecular Engineering, University of California, Irvine, CA 92697-3958, USA

## Abstract

Xeno-nucleic acids (XNAs) have gained significant interest as synthetic genetic polymers for practical applications in biomedicine, but very little is known about their biophysical properties. Here, we compare the stability and mechanism of acid-mediated degradation of α-l-threose nucleic acid (TNA) to that of natural DNA and RNA. Under acidic conditions and elevated temperature (pH 3.3 at 90°C), TNA was found to be significantly more resistant to acid-mediated degradation than DNA and RNA. Mechanistic insights gained by reverse-phase HPLC and mass spectrometry indicate that the resilience of TNA toward low pH environments is due to a slower rate of depurination caused by induction of the 2′-phosphodiester linkage. Similar results observed for 2′,5′-linked DNA and 2′-*O*-methoxy-RNA implicate the position of the phosphodiester group as a key factor in destabilizing the formation of the oxocarbenium intermediate responsible for depurination and strand cleavage of TNA. Biochemical analysis indicates that strand cleavage occurs by β-elimination of the 2′-phosphodiester linkage to produce an upstream cleavage product with a 2′-threose sugar and a downstream cleavage product with a 3′ terminal phosphate. This work highlights the unique physicochemical properties available to evolvable non-natural genetic polymers currently in development for biomedical applications.

## Graphical Abstract

**Graphical Abstract:**
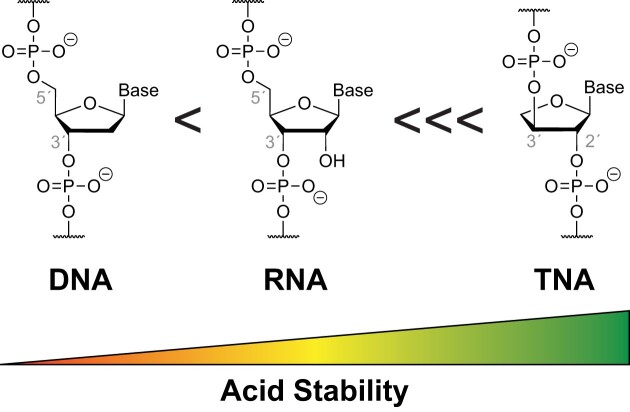

## INTRODUCTION

Chemical modifications are critical to the development of oligonucleotide therapeutics, as natural DNA and RNA oligonucleotides rapidly degrade in biological environments (1,2). In the last 50 years, hundreds of changes have been made to the sugar, base and phosphodiester backbone moieties of the molecule in an attempt to introduce molecular features that enhance the pharmacological properties of oligonucleotide therapeutics (3–6). This large body of work has significantly improved our chemical understanding of how changes in oligonucleotide structure correlate with improvements in target recognition, RNA hybridization, and nuclease stability (7–9). Although most studies have focused on relatively subtle chemical changes made to DNA and RNA, including 2′ substitutions (10), phosphodiester linkages (11), and alternative nucleobases (12,13), recent advances in nucleic acid chemistry have enabled the exploration of more diverse chemical architectures (14,15). Xeno-nucleic acids (XNA), artificial genetic polymers with backbone structures that are distinct from those found in nature (16), offer a promising new class of oligonucleotide therapeutics (17,18).

The unique physicochemical properties of XNA relative to natural DNA and RNA have attracted considerable attention as new materials for biomedicine (19,20). Favorable properties commonly attributed to XNAs include increased nuclease stability caused by their unnatural sugar-phosphate backbone structure and the potential for elevated thermodynamics of Watson–Crick base pairing. Locked nucleic acids (LNA), for example, are resistant to nuclease degradation and capable of increasing the melting temperature of DNA oligonucleotides by 4–8°C per residue when hybridized to complementary strands of RNA (21). XNAs are also thought to exhibit enhanced chemical stability, but detailed mechanistic studies evaluating their resilience under extreme conditions are lacking. One exception is 2′-fluoroarabino nucleic acid (FANA), which showed no signs of degradation after 2 days of incubation at 37°C in simulated gastric fluid (pH 1.2), under which conditions DNA and RNA degraded with half-lives (*t*_1/2_) of 2 min and 3 h, respectively (22). Since acid stability is known to be important for therapeutic delivery, especially endosomal escape (23), biochemical assays evaluating the mechanism and potential resistance to low pH environments are needed to guide the discovery of future XNA-based reagents.

**Figure 1.**
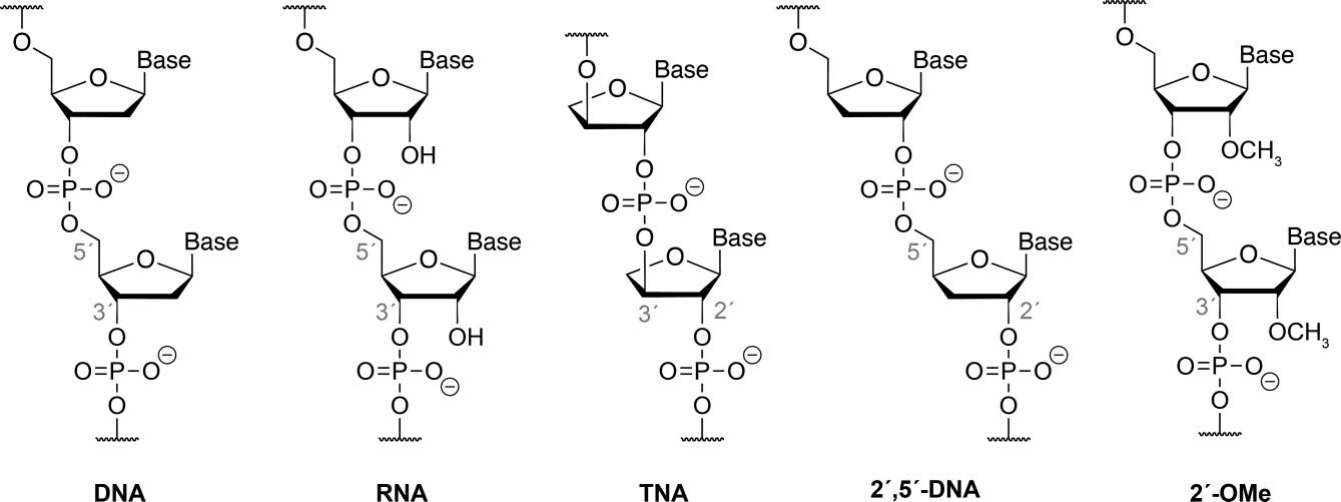
Chemical structures of the linearized backbones of DNA, RNA, TNA, 2′,5′-linked DNA, and 2′-methoxy RNA. α-l-threose nucleic acid (TNA) has one less atom in its backbone unit than DNA and RNA and shares an electron rich group at the 2′ position with 2′,5′-DNA and 2′-methoxy RNA.

Here we examine the stability and mechanism of acid-mediated degradation of a specific type of XNA known as α-l-threose nucleic acid (TNA, Figure 1) (24). TNA has received significant interest as an evolvable artificial genetic polymer due to its 2′,3′-phosphodiester linked backbone structure, which is refractory to nuclease digestion (25), yet still capable of forming stable antiparallel Watson-Crick duplex structures with complementary strands of DNA and RNA (24,26). Time course studies performed under acidic conditions and elevated temperature (pH 3.3 at 90°C) reveal that TNA is noticeably more pH stable than equivalent strands of natural DNA and RNA. Using 2′,5′-linked DNA and 2′-*O*-methoxy-RNA (OMe), we show that the enhanced stability of TNA to low pH environments is due to the position of the 2′-phosphodiester linkage, which destabilizes formation of the oxocarbenium intermediate responsible for depurination and subsequent strand cleavage. Further biochemical studies indicate that depurination is rate limiting and that strand cleavage proceeds by β-elimination of the 2′-phosphodiester linkage as opposed to β-elimination of the 3′-phosphodiester linkage observed in DNA. These findings offer new insights into the beneficial properties of XNAs as they apply to future applications in XNA therapeutics.

## MATERIALS AND METHODS

### Phosphoramidite preparation

DNA, 2′,5′-linked DNA, and 2′-OMe RNA phosphoramidite monomers were purchased from Glen Research. TNA phosphoramidites with standard bases were obtained by chemical synthesis as previously described (27,28). The abasic TNA phosphoramidite with a photocleavable 1-(2-methyl)nitrophenethyl (NPE) group was obtained by chemical synthesis as detailed in the Supplementary Information. All nonaqueous reactions were performed using oven-dried glassware under inert gas atmosphere. All chemicals and solvents were of laboratory grade as obtained from commercial suppliers and were used without further purification. Thin-layer chromatography (TLC) was performed on TLC aluminum sheets covered with silica gel 60 F254 (Sigma-Aldrich, St. Louis, Missouri). Flash column chromatography (FC): SiliCycle 40–60 mesh silica gel (SiliCycle Inc., Quebec City, Canada). Yields are reported as isolated yields of pure compounds. ^1^H, ^13^C and ^31^P NMR spectra were obtained using Bruker DRX400 NMR spectrometer (Bruker, Billerica, Massachusetts). δ values in ppm relative to Me_4_Si or corresponding deuterium solvents as internal standard (^1^H and ^13^C). ^31^P NMR values are reported in ppm relative to an external standard of 85% H_3_PO_4_.

### Oligonucleotide preparation

All oligonucleotides (Table S1) were synthesized in-house by solid-phase oligonucleotide synthesis on a 1 μmol scale, deprotected by 28% aqueous ammonium hydroxide for 18 h at 55°C, and purified by using an oligonucleotide purification cartridge (OPC) and RP-HPLC, except for the RNA oligonucleotides and DNA standards, which were ordered from Integrated DNA Technologies (IDT). Oligonucleotides ordered from IDT were used without further purification. NPE-protected TNA oligonucleotides were dissolved in 200 μl of water to a final concentration of 200 μM and deprotected at room temperature using an Analytik Jena UVP Crosslinker CL-1000L at an energy setting of 80 mJ/cm^2^ for 7 min. Glen-Pak™ DNA Purification Cartridges were purchased from Glen Research. The Zorbax SB-C18 HPLC column (9.4 mm × 250 mm, 5 mM) for semi-preparative HPLC and Discovery C18 HPLC column (4.6 mm × 10 cm, 5 mM) for analytical HPLC were purchased from Agilent and Millipore Sigma, respectively.

### Acid stability assay

Oligonucleotides were dissolved to a final concentration of 40 μM in 120 mM citrate phosphate buffer (pH 3.3) in a total reaction volume of 100 μl and heated at 90°C on a pre-heated heat block. At designated time points, the reactions were removed from the heat block, briefly vortexed, and allowed to cool (∼2 min). In each case, aliquots of 20 μl were then taken from the reaction samples and quenched in 80 μl of 50 mM triethylammonium bicarbonate (TEAB) (pH 8.5) to a final concentration of 8 μM for analysis by RP-HPLC.

### Acid stability analysis by RP-HPLC

RP-HPLC analysis was performed on a Discovery C18 HPLC column with buffer A as 50 mM TEAB (pH 8.5) and buffer B as acetonitrile. An increasing gradient of 0% to 20% buffer B was generated over 14 min with a flow rate of 1.00 ml/min at a set temperature of 40°C. The percent full-length product (%FLP) was determined as the relative area percentage under the absorbance curve at 260 nm calculated by the Chromeleon Chromatography Data System (CDS) Software. The %FLP at each time point was then divided by the %FLP at time point *t* = 0 and multiplied by 100, normalizing the %FLP at *t* = 0 to 100. The normalized values were then graphed in LabPlot2 and fitted to the exponential function (*y* = *a*e^*bx*^) to calculate the half-lives. Additionally, ln[*S*]/[*S*_0_] was plotted versus time and fit to a linear function of first order kinetics, where [*S*]/[*S*_0_] is the ratio of the remaining FLP to the starting FLP as determined by HPLC peak integration at the specified time points. The rate constants for acid-mediated degradation were then obtained from the slope of the linear fit.

### Mechanistic study of acid-mediated degradation

The oligonucleotide sequence used to study the degradation mechanism was 5′/3′-TTT TTT ATT TTT TTT T-3′/2′. The asymmetric oligonucleotide design (T_6_ versus T_9_) allowed for easy differentiation of the cleavage products by mass spectrometry. The depurination assay was performed in a manner identical to the acid stability assay with sample collection either up to the time point when the FLP was mostly degraded or 24 h.

### Acid-mediated degradation analysis by MALDI-TOF

The samples collected from the mechanistic study using the asymmetric sequence were desalted with ZipTip C18 pipette tips and pipetted directly onto a MALDI-TOF steel target plate (∼1 μl). The spot on the plate was then dried by briefly heating at 55°C (∼2 min) and covered with 1 μl of matrix (18 mg/ml 2,4,6-trihydroxy-acetophenone (THAP) and 6 mg/ml dibasic ammonium citrate in HPLC grade 50:50 MeCN/water) for characterization on a Bruker Daltronics Microflex MALDI-TOF mass spectrometer.

### Molecular dynamics setup and simulations

A BespokeFit workflow was utilized using OpenFF Toolkit (https://github.com/openforcefield/openff-toolkit/tree/0.7.2) and OpenEye tools (OpenEye Scientific Software, Inc.) to generate and parameterize a force field for a ring-opened aldehyde TNA monomer using xTB (gfn2xtb) (27,28). OpenFF Interchange was used to create the initial topology and coordinates (https://github.com/openforcefield/openff-interchange). Molecular dynamics simulations were prepared and ran using GROMACS (v2022.1; gcc.8.4.0) as described by Lemkul, with discrepancies indicated subsequently (29,30). In brief, the molecule was solvated in a cubic water box (spc216) with periodic boundary conditions in three dimensions and a buffering distance of 1 nm for a total of 856 water molecules treated using the SETTLE algorithm (31). A 50 000-step steepest descent energy minimization was performed on the monomer plateauing at −7.64e + 05 kcal/mol. Initial velocities were generated from a Maxwell distribution at 363 K. The system was equilibrated in a canonical ensemble (NVT) and then an isobaric-isothermic ensemble (NPT) for 100 ps each at 363 K to stabilize the system. The production run was done for 10 ns, with a time step of 2 fs. Replicates of the simulation were performed starting from the same initial topology and coordinates created using Interchange. Dihedral angles were analyzed from trajectories using a custom script in PyMOL (v2.0, Schrödinger, LLC.) and visualized using Matplotlib (32).

## RESULTS AND DISCUSSION

**Figure 2.**
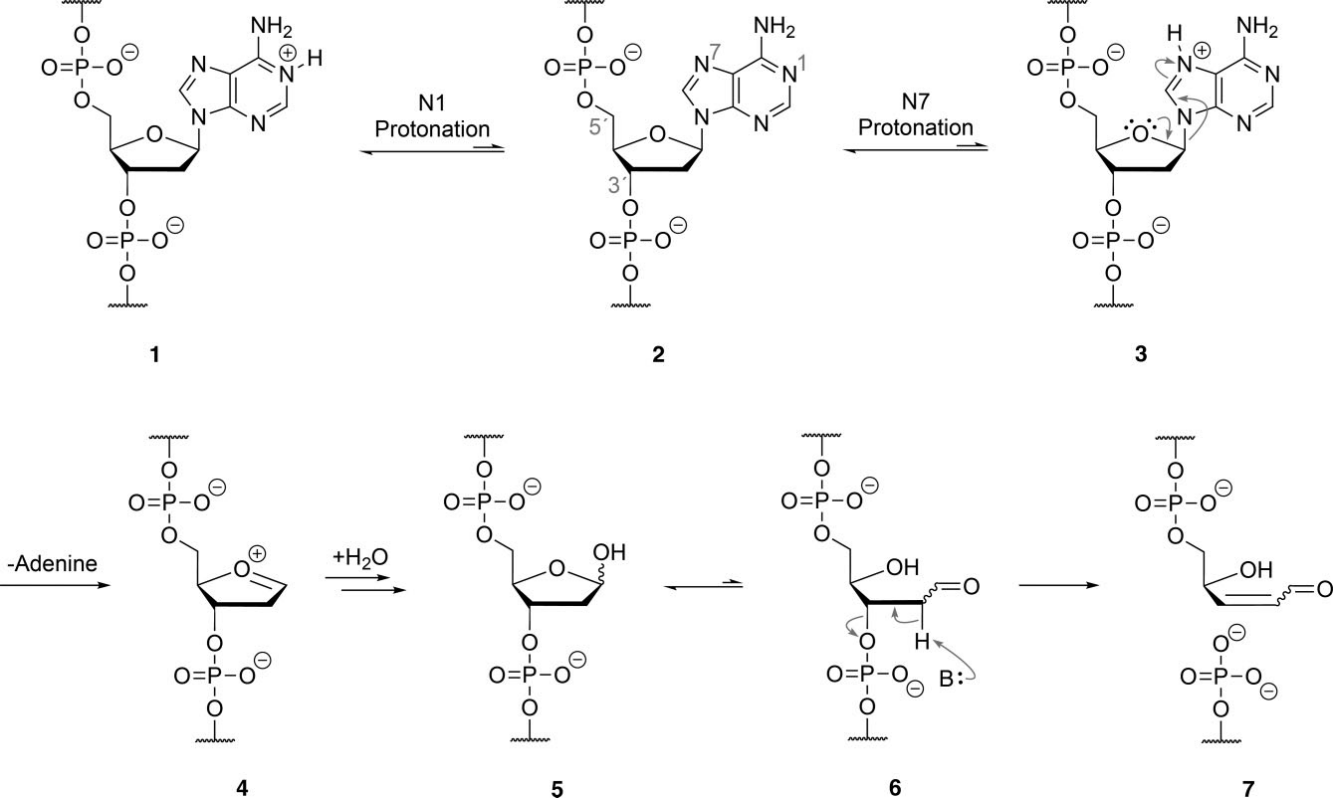
General mechanism of acid-mediated degradation of DNA. Under acidic conditions, purine **2** is protonated at its N7 position, weakening the *N*-glycosidic bond of **3** and generating an abasic site after elimination of purine. A hemiacetal **5** is formed in equilibrium with its ring-opened aldehyde **6** form after hydration of the oxocarbenium ion **4**. Strand scission occurs by an E2 elimination to produce an upstream cleavage product with a 3′-terminal aldehyde (**7**, top) and a downstream cleavage product with a 5′-terminal monophosphate (**7**, bottom).

The mechanism of acid-mediated DNA strand cleavage has been known for more than 50 years (33), and the susceptibility of RNA therapeutics to chemical degradation in low pH environments remains an important part of the drug discovery process (34). As illustrated in Figure 2, the exposure of DNA to low pH environments facilitates protonation of purine nucleotides at the *N*7 position **2**, and subsequent depurination via cleavage of the glycosidic bond **3**. However, we wish to point out that the first protonation site on adenosine occurs at the *N*1 position **1**, but this step is generally excluded from the mechanism because it does not weaken the glycosidic bond to the extent as protonation at the *N*7 position (35). The addition of water to the resulting oxocarbenium intermediate **4** of the abasic deoxyribose sugar leads to the formation of hemiacetal **5** that resides in equilibrium (99:1) with its ring-opened aldehyde form **6** (36). Despite its low abundance, aldehyde **7** is particularly sensitive to strand scission through an E2 elimination reaction that occurs via deprotonation of the proton alpha to the aldehyde group. In the case of RNA, acid-mediated strand cleavage occurs via attack of the 2′ hydroxyl on the protonated phosphate group (37). However, at pH values less than 3, acid-catalyzed depurination competes with phosphate hydrolysis (38), noting that depurination is slower for RNA than DNA due to the electronegative oxygen atom at the 2′ position, which inductively destabilizes the oxocarbenium ion intermediate (39,40). Substitution of the 2′ hydroxyl group for a more electronegative atom like, fluorine, further destabilizes the oxocarbenium intermediate, reducing its susceptibility to strand cleavage by depurination, as seen in 2′-fluorinated RNA (F-RNA) (41). This effect also accounts for the enhanced acid stability observed in FANA (22).

Based on the mechanism of acid-mediated DNA strand cleavage, we predicted that TNA would share a similar destabilizing effect on the oxocarbenium ion intermediate responsible for depurination as previously observed for F-RNA and FANA (22,41). To explore this prediction in greater detail, we compared the pH stability of a randomly generated 24 nucleotide (nt) sequence prepared as DNA, RNA, TNA and 2′,5′-linked DNA (Figure 1). Initial studies were performed using the DNA and TNA molecules to evaluate a range of conditions, including varying the pH, temperature and incubation time. Since no degradation was observed for solutions held at 37°C (pH 4) for up to 48 h, a full pH range of 4–10 was explored at 55°C. Under these conditions, TNA remained intact, while the DNA molecule showed noticeable signs of degradation after 24 h of incubation at pH 4 (Supplementary Figure 1). For feasibility reasons, all subsequent stability studies were performed in 120 mM citrate phosphate buffer (pH 3.3) at 90°C, which allowed acid-mediated degradation to be monitored on a reasonable timescale. At designated time points, aliquots were removed, quenched with 0.05 M triethylammonium bicarbonate (TEAB) (pH 8.5), and directly analyzed by ion pair reverse phase high pressure liquid chromatography (RP-HPLC).

**Figure 3.**
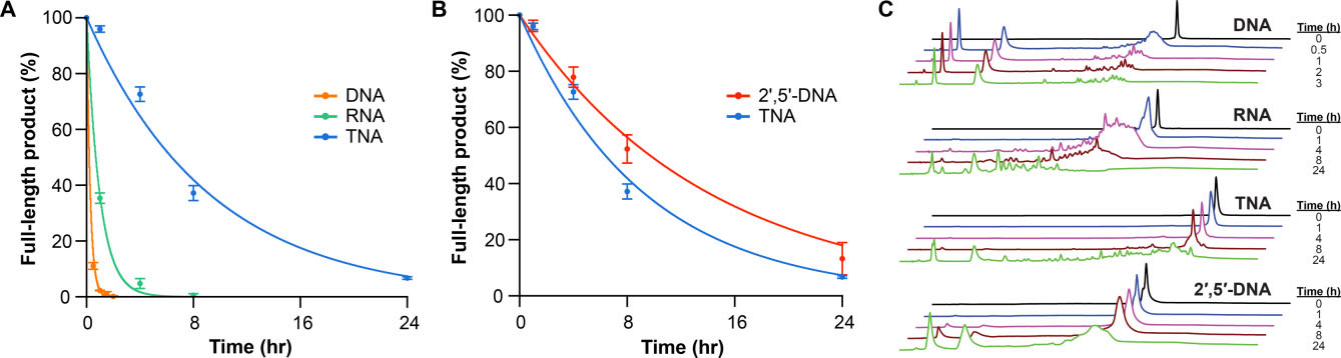
Time-dependent acid stability. (**A**, **B**) Acid-mediated oligonucleotide decay curves. Degradation profiles of DNA, RNA, TNA and 2′,5′-DNA oligonucleotides prepared with the same nucleotide sequence (5′/3′-CCGTAGTGAAAGATCCCTGTTCAG-3′/2′) were monitored as a function of time by RP-HPLC. Error bars denote ± standard deviation of the mean for three independent replicates. All reactions were performed in 120 mM citrate phosphate buffer (pH 3.3) poised at 90°C. (**C**) Representative reverse phase HPLC chromatograms observed for each genetic system show oligonucleotide degradation as a function of time.

In the low pH high temperature environment, TNA was found to be more resistant than DNA and RNA toward acid-mediated chemical degradation. Half-life values (*t*_1/2_) of 10.9 and 40.8 min were observed for DNA and RNA, which correspond to rate constants of 8.4 × 10^−4^ s^−1^ and 1.9 × 10^−4^ s^−1^, respectively (Figure 3A, Supplementary Figure 2). By comparison, TNA with its unusual 2′,3′-linked phosphodiester backbone, remained largely unmodified (>90% of full-length product) after 1 h of incubation with a calculated *t*_1/2_ value of 6.3 h and a rate constant of 3.3 × 10^−5^ s^−1^ (Figure 3A, Supplementary Figure 2). Postulating that the observed acid stability of TNA was due to the location of the phosphodiester group at the 2′ position as opposed to the more common 3′ position found in DNA and RNA, we chose to compare TNA to a naturally occurring analog of DNA known as 2′,5′-linked DNA (42), a product of the interferon regulated synthesis of 2′,5′-oligoadenylate (43). Under the low pH and high temperature conditions, 2′,5′-linked DNA exhibits a *t*_1/2_ value of 9.9 h with an observed rate constant of 2.5 × 10^−5^ s^−1^ (Figure 3B, Supplementary Figure 2), making it slightly more stable than TNA against acid-mediated degradation. Based on these results, the order of increasing stability observed for our randomly generated 24 nt sequence is DNA < RNA << TNA < 2′,5′-DNA as visualized by RP-HPLC analysis (Figure 3C).

The enhanced stability observed for TNA and 2′,5′-linked DNA strongly supports the hypothesis that the location of the phosphodiester linkage is important for stabilizing both genetic systems against acid-mediated chemical degradation. Although we suspect that the chemical underpinnings of the enhanced acid stability are due to destabilization of the oxocarbenium ion responsible for adenosine depurination and strand cleavage, we cannot discount the possibility that other steric or electronic effects may be contributing to the observed stability of TNA and 2′,5′-linked DNA in a low pH environment. In an effort to gain deeper insight into the mechanism of acid-mediated degradation, we designed an asymmetric oligonucleotide sequence (T_6_AT_9_) that contained a central adenosine residue flanked by six thymidine residues on the 5′ side and nine thymidine residues on the 3′ side of the purine. The asymmetric design made it possible to follow the degradation pathway by standard analytical techniques of RP-HPLC and mass spectrometry. Oligonucleotides prepared with the T_6_AT_9_ sequence were incubated for various times in 120 mM citrate phosphate buffer (pH 3.3) at 90°C. Aliquots collected at desired times of the decay assay were quenched with 0.05 M TEAB (pH 8.5) and directly analyzed by RP-HPLC. Prior to HPLC analysis, a small portion of the sample was removed for mass spectrometry analysis.

**Figure 4.**
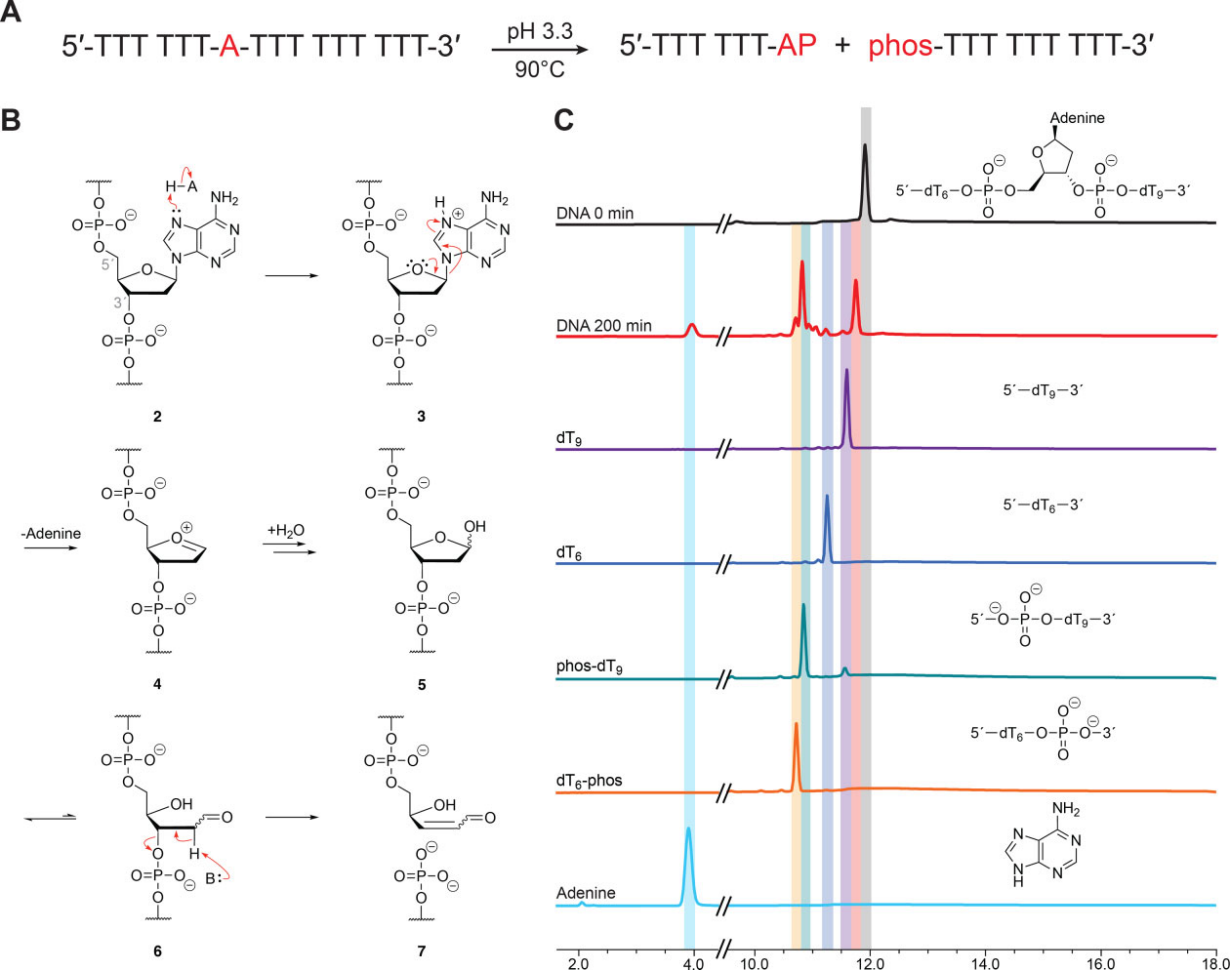
Mechanism of acid-mediated DNA strand cleavage. (**A**) Asymmetric oligonucleotide design. Acid-mediated cleavage results in an upstream cleavage product with a 3′ terminal abasic site (AP) and a downstream product with a 5′-monophosphate (phos). (**B**) Degradation involves the formation of an abasic strand that undergoes ring opening and strand cleavage via E2 elimination of the 3′-phosphodiester linkage. (**C**) Representative reverse phase HPLC chromatograms comparing the 5′-d(T_6_AT_9_)-3′ strand before and after 200 min of acid treatment to authentic standards prepared for key intermediates.

Upon exposure to acid, we predicted that the asymmetric DNA oligonucleotide would degrade into two main cleavage products, 5′-dT_6_-AP-3′ and 5′-phos-dT_9_-3′ (Figure 4A, where AP signifies the depurinated residue), which is consistent with the classic E2 elimination mechanism proposed for acid-mediated DNA strand cleavage (Figure 4B) (33,34). After 200 min of incubation at 90°C (pH 3.3), two large peaks were observed in the RP-HPLC traces, along with several smaller peaks (Figure 4C, Supplementary Figure 3). Mass spectrometry analysis identified the two large peaks as the depurinated form of the T_6_AT_9_ strand and the downstream cleavage product of 5′-phos-dT_9_-3′ (Supplementary Figure 4). The depurinated T_6_AT_9_ intermediate was detected as the dehydrated form, which is likely due to MALDI-TOF analysis. Most of the smaller peaks observed in the RP-HPLC trace were identified using a combination of authentic standards and mass spectrometry (Figure 4C, Supplementary Figure 4). The smaller peaks are primarily the result of further degradation of the 5′-dT_6_-AP-3′ and 5′-phos-dT_9_-3′ fragments, including the 5′-dT_6_-phos-3′ and 5′-dT_6_-3′ intermediates from the upstream cleavage product and the 5′-dT_9_-3′ intermediate from the downstream cleavage product. Although the upstream product (5′-dT_6_-AP-3′) was not observed by HPLC, this intermediate was detected by mass spectrometry (Supplementary Figure 4). Interestingly, a control experiment performed with 5′-phos-dT_9_-3′ confirmed that dephosphorylation occurs under the low pH conditions (Supplementary Figure 5), indicating that multiple different hydrolysis reactions are taking place as the initial cleavage products undergo further decay. Time-dependent studies show that depurination is rapid for the asymmetric DNA sequence (Supplementary Figure 3), suggesting that adenine departure is the fast step of acid-mediated DNA degradation.

**Figure 5.**
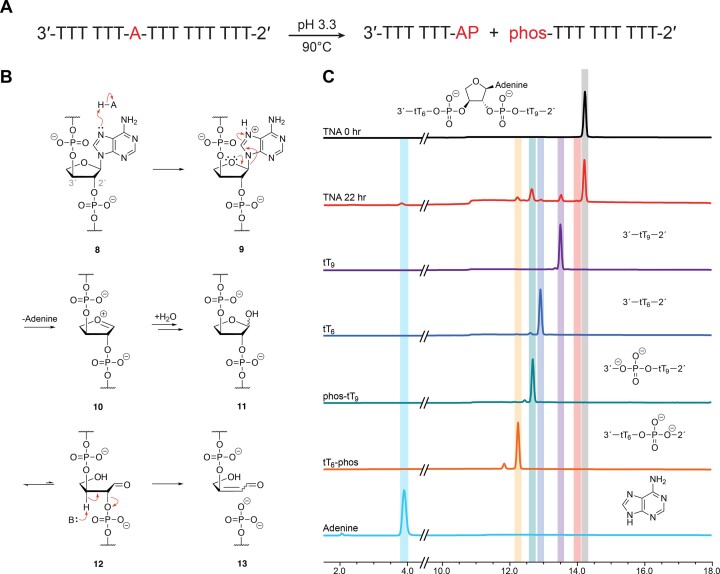
Mechanism of acid-mediated TNA strand cleavage. (**A**) Asymmetric oligonucleotide design. Acid-mediated cleavage results in an upstream cleavage product with a 2′-terminal abasic site (AP) and a downstream product with a 3′-monophosphate (phos). (**B**) Degradation involves the formation of an abasic strand that undergoes ring opening and strand cleavage via β-elimination of the 2′-phosphodiester linkage. (**C**) Representative reverse phase HPLC chromatograms comparing the 5′-t(T_6_AT_9_)-3′ strand before and after 22 h of acid treatment to authentic standards prepared for key intermediates.

A similar mechanistic study was performed on the asymmetric sequence prepared as TNA. This genetic system was more difficult to analyze due to the presence of phosphodiester linkages at both the 2′ and 3′ positions of the sugar. As such, it was unclear whether strand scission would occur via elimination of the 3′ phosphodiester linkage, as is commonly observed for DNA and RNA, or through a novel degradative pathway involving elimination of the 2′ phosphodiester linkage (Figure 5A-B). Time-dependent HPLC traces collected over a 22-h period of incubation at 90°C (pH 3.3) yielded the full-length T_6_AT_9_ sequence with several smaller peaks (Figure 5C). RP-HPLC comparisons performed with authentic standards (Figure 5C) identified two of the smaller peaks as the phosphorylated and dephosphorylated forms of the downstream 2′ cleavage product (3′-phos-tT_9_-2′ and 3′-tT_9_-2′). We confirmed that the 3′-tT_9_-2′ intermediate can form under the current conditions by evaluating the decay profile for an authentic standard of 3′-phos-tT_9_-2′ prepared by solid-phase synthesis (Supplementary Figure 5B).

To better understand the mechanism of TNA degradation, we used mass spectrometry to identify additional intermediates that were present at levels below the analytical limit of detection for HPLC analysis. In addition to the downstream intermediates previously identified by RP-HPLC, mass spectrometry confirmed the presence of various upstream degradative products including the abasic derivative, 3′-tT_6_-AP-2′ and its deglycosylated form (Supplementary Figure 6). This result implies that acid-mediated TNA degradation primarily occurs via elimination of the 2′ phosphodiester linkage with depurination being the rate limiting step of degradation (Figure 5A, B). Additionally, we also observe what appears to be evidence of a competing *syn*-elimination pathway via addition of the anomeric hydroxyl group to the 2′-phosphodiester linkage (Supplementary Figure 7), resulting in a product with an observed mass of 1961.4 (Supplementary Figure 6). However, given the small size of this peak in the abasic TNA control assay (see Supplementary Figure 9), we postulate that *syn*-elimination is a minor pathway as compared to β-elimination.

**Figure 6.**
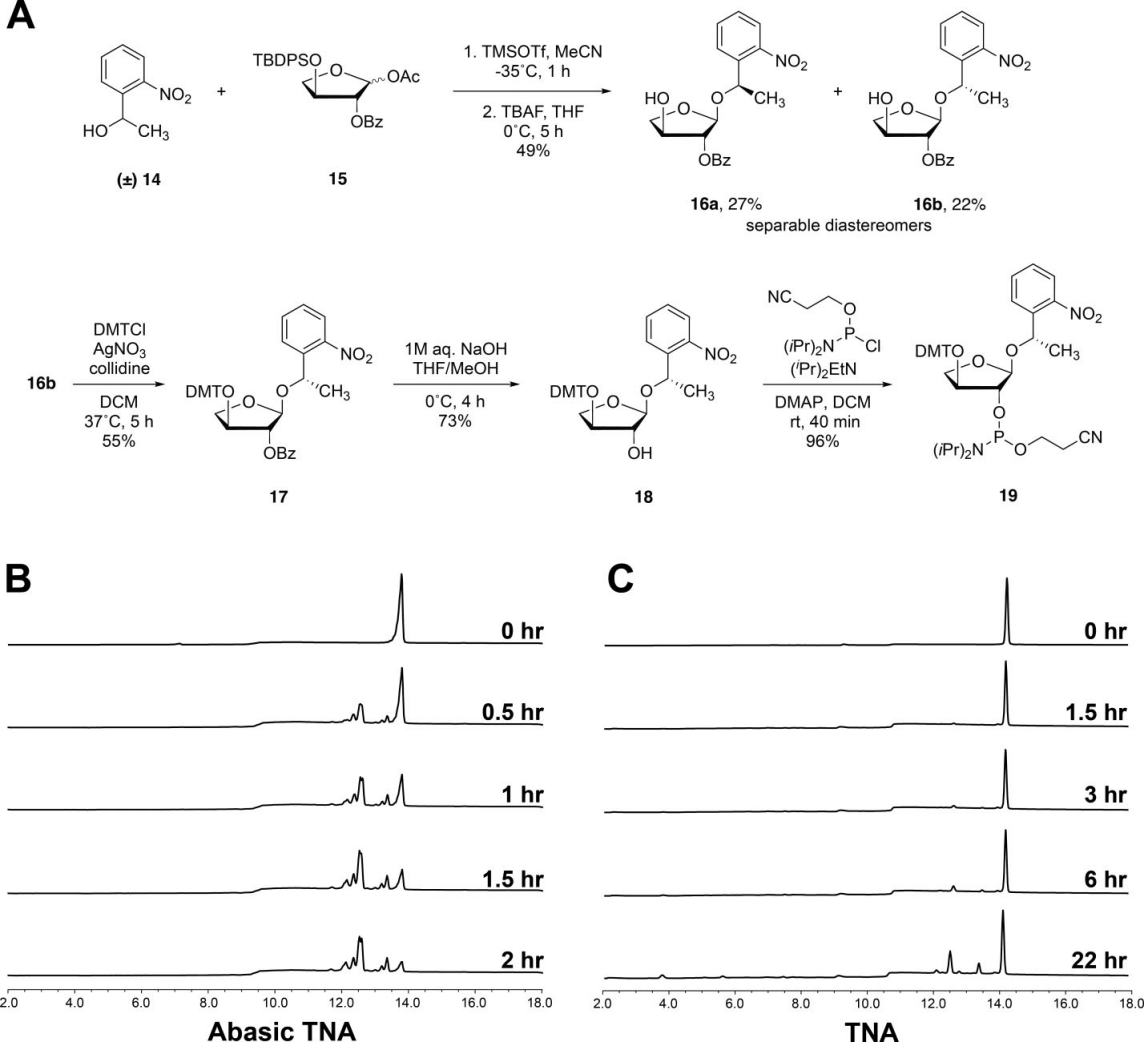
Synthesis and evaluation of an abasic TNA toward acid-mediated TNA degradation. (**A**) Reaction scheme used to prepare the abasic TNA phosphoramidite. (**B**, **C**) Time-dependent HPLC analysis of acid-mediated degradation of an abasic TNA strand (tT_6_-tAP-tT_9_, where tAP signifies the abasic residue) versus an all-TNA strand (tT_6_-tA-tT_9_). The abasic TNA strand shows faster degradation kinetics than the all-TNA strand under the same reaction conditions (pH 3.3 at 90°C).

To further evaluate the cleavage mechanism, we prepared and analyzed the abasic intermediate (tT_6_-tAP-tT_9_, where tAP signifies the abasic residue) that arises following depurination of the asymmetric TNA strand. The TNA oligonucleotide was synthesized using a novel abasic TNA phosphoramidite that was prepared by chemical synthesis following methodology that was previously established for RNA (Figure 6A) (44). The abasic phosphoramidite **19** was prepared from racemic 1-(2-nitrophenyl)ethan-1-ol **14** as the glycosyl acceptor and a fully protected α-L threofuranosyl sugar **15** as the glycosyl donor. In presence of trimethylsilyl trifluoromethanesulfonate (TMSOTf) in CH_3_CN at −35°C, the reaction produced a diastereomeric mixture of glycosylated products. After removal of the 3′-O-TBDPS group, the glycosylated products were separated by silica gel column chromatography to give **16a** and **16b** in a total yield of 49% for the two steps. The structure of **16a** was confirmed by comparing the ^1^H NMR of the corresponding product obtained from enantiopure (*R*)-1-(2-nitrophenyl)ethan-1-ol. Since we were not concerned about the stereochemistry, compound **16b** was selected based on purity and converted to the desired phosphoramidite **19** by protection of 3′-OH group with 4,4′-dimethoxytritylchloride (DMTCl), removal of the 2′ benzoyl group with 1M NaOH, and treatment of **18** with 2,2-cyanoethyl-*N*,*N*-diisopropylchlorophosphoramidite in the presence of Hünig's base. The TNA phosphoramidite **19** was then incorporated into the asymmetric sequence by solid-phase synthesis, and the final abasic TNA oligonucleotide was obtained after deprotection of the NPE group by UV irradiation (Supplementary Figure 8).

Time-dependent HPLC and mass spectrometry analysis of the abasic TNA strand indicate that strand cleavage is complete by 2 h (Figure 6B), while the equivalent adenosine-containing TNA strand remains mostly undegraded (>90%) after 22 h of incubation (Figure 6C). The difference in the rates of degradation between the abasic control and standard base TNA strand confirms that depurination is the rate limiting step of acid-mediated TNA degradation. Mass spectrometry analysis of the abasic strand identified the expected cleavage products, 3′-tT_6_-AP-2′ and 3′-phos-tT_9_-2′, along with the same minor products previously observed for the adenosine-containing strand (Supplementary Figure 9). Although the abasic TNA strand contained trace amounts of the n-1 and n-2 as byproducts from solid-phase synthesis, their presence did not affect the results of the assay. In fact, MALDI-TOF analysis showed that the n-1 byproduct degrades in a manner similar to the full-length product (Supplementary Figure 9). Importantly, mass spectrometry analysis did not identify the 3′-AP-tT_9_-2′ intermediate, providing further evidence for strand cleavage via β-elimination of the 2′-phosphodiester linkage.

Additional control experiments were performed to compare the acid stability observed for TNA to other DNA and RNA analogs. For these experiments, we prepared the asymmetric sequence with a central adenosine residue carrying either a 2′-5′-linkage or a 2′-OMe modification. Both controls yield stability profiles similar to the all-TNA version of the asymmetric sequence with depurination being the rate limiting step in each case (Supplementary Figures 3, 10) (45). Similar results were also observed for a fully modified version of the 2′-OMe sequence (Supplementary Figure 3). Collectively, this data supports the conclusion that the enhanced acid stability of TNA is due to the position of the phosphodiester group in the nucleic acid backbone. This information, along with other biophysical properties, like nuclease stability, should be useful in the design of future XNA therapeutics.

**Figure 7.**
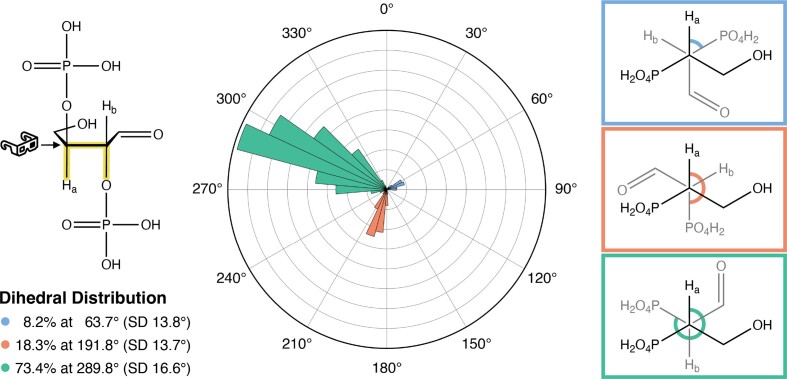
Conformational sampling via molecular dynamics. The dihedral distribution about the highlighted bond (H–C3'–C2'–O) is plotted following a 10 ns molecular dynamics simulation of the ring-open aldehyde TNA fragment in water. Newman projections are depicted for angles 60°, 180° and 300°. Additional replicates of the 10 ns simulation are reported in Supplementary Figure 11.

In an effort to gain insight into the regioselectivity of the elimination mechanism in TNA, we evaluated a TNA monomer in its ring-opened aldehyde form in water at 363 K (90°C) via 10 ns molecular dynamics simulations. Torsion analysis about the C3'–C2' bond revealed that protons HC3' and HC2' (denoted H_a_ and H_b_, respectively) are virtually antiperiplanar to their corresponding phosphates during 22.9 ± 2.7% of the time sampled (Figure 7, Supplementary Figure 11). While feasible, both protons are concomitantly *anti* to the corresponding phosphates with no clear structurally favorable path of elimination. While we cannot exclude possible steric and electronic effects of the full oligonucleotide strand on the preferred conformation for elimination, it is evident that there is preference toward elimination of the 2′ phosphodiester linkage based on our analytical HPLC and mass spectrometry data.

## CONCLUSION

In summary, our results demonstrate that TNA is significantly more resistance to acid-mediated degradation than natural DNA and RNA. Mechanistic studies suggest that the enhanced stability is due to inductive destabilization of the oxocarbenium intermediate, which is analogous to previous studies on 2′-methoxy-RNA and consistent with our control experiments on 2′,5′-DNA. We further show that acid-mediated strand cleavage in TNA occurs via β-elimination of the 2′-phosphodiester linkage rather than the 3′-phosphodiester linkage observed for DNA, illustrating the complexity of the chemistry behind the properties of artificial genetic polymers. These observations offer further insight into the unique biophysical properties of XNAs and provide further support for expanding the use of artificial genetic polymers in biomedical applications involving extreme conditions that are not suitable for DNA and RNA.

## Supplementary Material

gkad716_Supplemental_File

## Data Availability

The data underlying this article will be shared on reasonable request to the corresponding author.
