# Supplementary material for: Stability and mechanism of threose nucleic acid toward acid-mediated degradation
Source: Nucleic Acids Res. 2023 Aug 31;51(18):9542–51. doi: 10.1093/nar/gkad716 (PMC10570051; doi:10.1093/nar/gkad716)
Supplement: gkad716_Supplemental_File [file gkad716_supplemental_file.pdf]

Supplementary Information

Stability and Mechanism of Threose Nucleic Acid Toward Acid-Mediated Degradation

Erica M. Lee,[1] Noah A. Setterholm,[1] Mohammad Hajjar,[1] Bhawna Barpuzary,[1] and John C. Chaput[1-4]*

[1]Department of Pharmaceutical Sciences, University of California, Irvine, CA 92697-3958, United States
[2]Department of Chemistry, University of California, Irvine, CA 92697-3958, United States
[3]Department of Molecular Biology and Biochemistry, University of California, Irvine, CA 92697-3958, United States
[4]Department of Chemical and Biomolecular Engineering, University of California, Irvine, CA 92697-3958, United States

Table of Contents

Table S1. List of oligonucleotides.

Figure S1. Preliminary assessment of the stability of DNA versus TNA under varying pH and temperature conditions.

Figure S2. Linear plots of $\ln[S]/[S_0]$ versus time.

Figure S3. Time-dependent HPLC analysis of acid-mediated cleavage of the asymmetric control strands.

Figure S4. Time-dependent mass spectrometry analysis of acid-mediated cleavage of DNA.

Figure S5. Time-dependent RP-HPLC and mass spectrometry analysis of acid hydrolysis of phosphorylated DNA and TNA.

Figure S6. Time-dependent mass spectrometry analysis of acid-mediated cleavage of TNA.

Figure S7. Elimination pathways in acid-mediated TNA degradation.

Figure S8. HPLC analysis of NPE deprotection of asymmetric abasic TNA control strand via UV irradiation.

Figure S9. Time-dependent mass spectrometry analysis of acid-mediated cleavage of TNA with an abasic residue.

Figure S10. Time-dependent mass spectrometry analysis of acid-mediated cleavage of a chimeric DNA with a central 2',5'-linked adenosine residue.

Figure S11. Replication of conformational sampling.

Abasic TNA Phosphoramidite Synthesis.

Compound Characterization.

Supplementary Table 1. List of oligonucleotides.

#	Identifier	Sequence	Length (bp)	Calc. MW (g/mol)	Obs. MW (g/mol)
1	RandSeq2 DNA	5'-d(CCG TAG TGA AAG ATC CCT GTT CAG)-3'	24	7352.8	7354.5
2	RandSeq2 RNA	5'-r(CCG UAG UGA AAG AUC CCU GUU CAG)-3'	24	7652.6	7654.7
3	RandSeq2 TNA	3'-t(CCG TAG TGA AAG ATC CCT GTT CAG)-2'	24	7016.3	7016.9
4	RandSeq2 2-5	5'-d(CCG TAG TGA AAG ATC CCT GTT CAG)-2'	24	7352.8	7357.9
5	DNA T$_6$AT$_9$	5'-d(TTT TTT ATT TTT TTT T)-3'	16	4814.2	4814.9
6	TNA T$_6$AT$_9$	3'-t(TTT TTT ATT TTT TTT T)-2'	16	4589.7	4590.6
7	TNA T$_6$tAPT$_9$	3'-t(TTT TTT) tAP t(TT TTT TTT T)-2'	16	4472.6	4473.4
8	2',5' T$_6$AT$_9$	5'-d(TTT TTT) A d(TT TTT TTT T)-2'	16	4814.2	4812.4
9	2'-OMe U$_6$AU$_9$	5'-m(UUU UUU AUU UUU UUU U)-3'	16	5084.2	5085.3
10	2'-OMe T$_6$mAT$_9$	5'-d(TTT TTT) mA d(TT TTT TTT T)-2'	16	4844.2	4846.3
11	DNA dT$_9$	5'-d(TTT TTT TTT)-3'	9	2675.8	2674.1
12	DNA pdT$_9$	5'-phos-d(TTT TTT ATT TTT TTT T)-3'	9	2755.8	2755.3
13	DNA dT$_6$	5'-d(TTT TTT)-3'	6	1763.2	1763.0
14	DNA dT$_6$p	5'-d(TTT TTT)-phos-3'	6	1843.2	1842.7
15	TNA tT$_9$	3'-t(TTT TTT TTT)-2'	9	2549.5	2549.7
16	TNA ptT$_9$	3'-phos-t(TTT TTT ATT TTT TTT T)-2'	9	2629.5	2629.7
17	TNA tT$_6$	3'-t(TTT TTT)-2'	6	1679.0	1680.1
18	TNA tT$_6$p	3'-t(TTT TTT)-phos-2'	6	1759.0	1759.4

All masses were determined by MALDI-TOF mass spectrometry.
tAP bases are abasic TNA.
Underlined bases are 2',5'-linked DNA.
mN bases are 2'-OMe RNA.

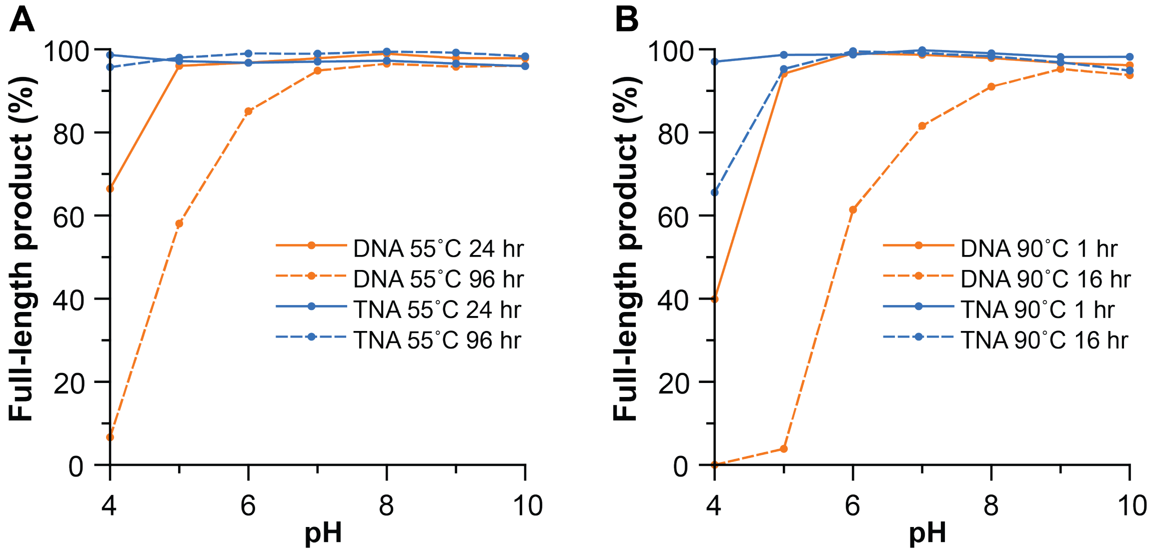

Supplementary Figure 1. Preliminary assessment of the acid stability of DNA and TNA under varying pH and temperature conditions. A) Stability profile of DNA and TNA observed across a pH range of 4 to 10 at 55°C. B) Stability profile of DNA and TNA observed across a pH range of 4 to 10 at 90°C.

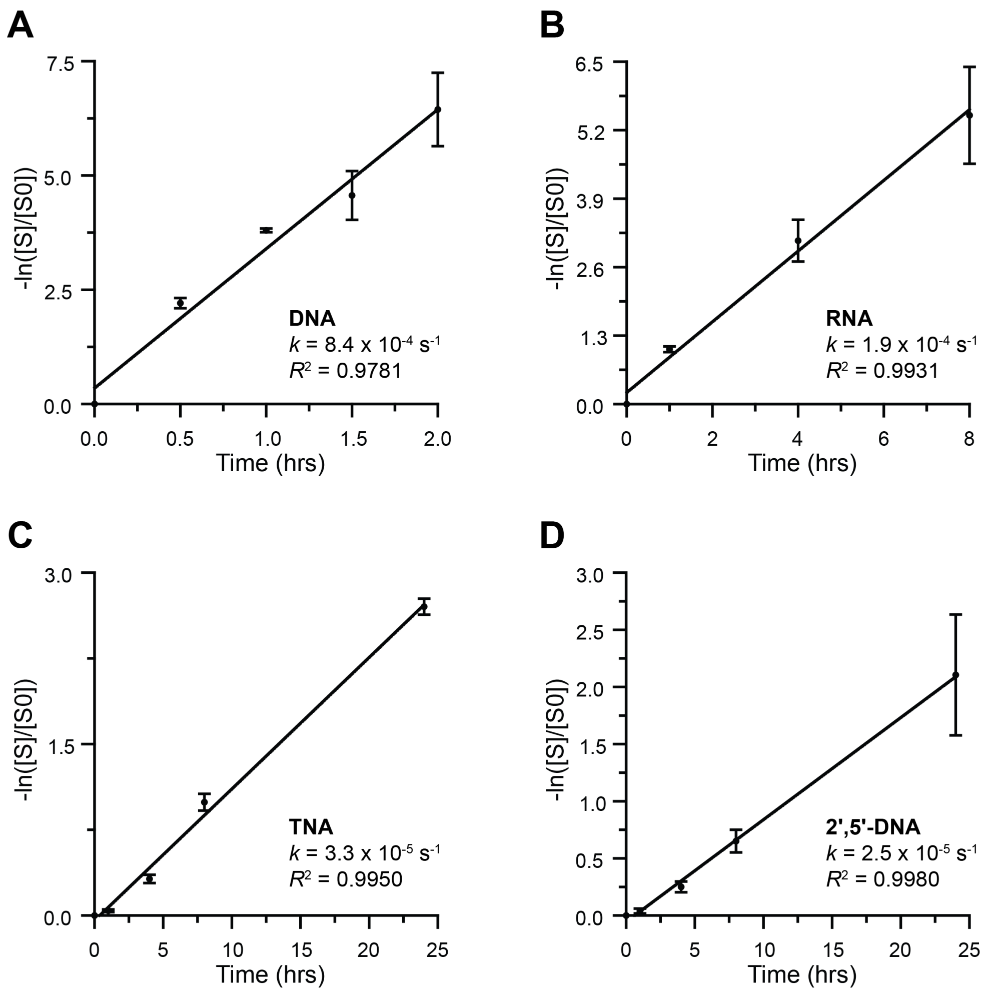

Supplementary Figure 2. Linear plots of ln[S]/[S_0] versus time. First order rate constants (*k*) for acid-mediated degradation of DNA (A), RNA (B), TNA (C), and 2',5'-DNA (D) were obtained from the slope of the depicted linear plots, where [S]/[S_0] is the ratio of the remaining FLP to the starting FLP as determined by HPLC peak integration at the specified time points. Error bars denote ± standard deviation (n=3).

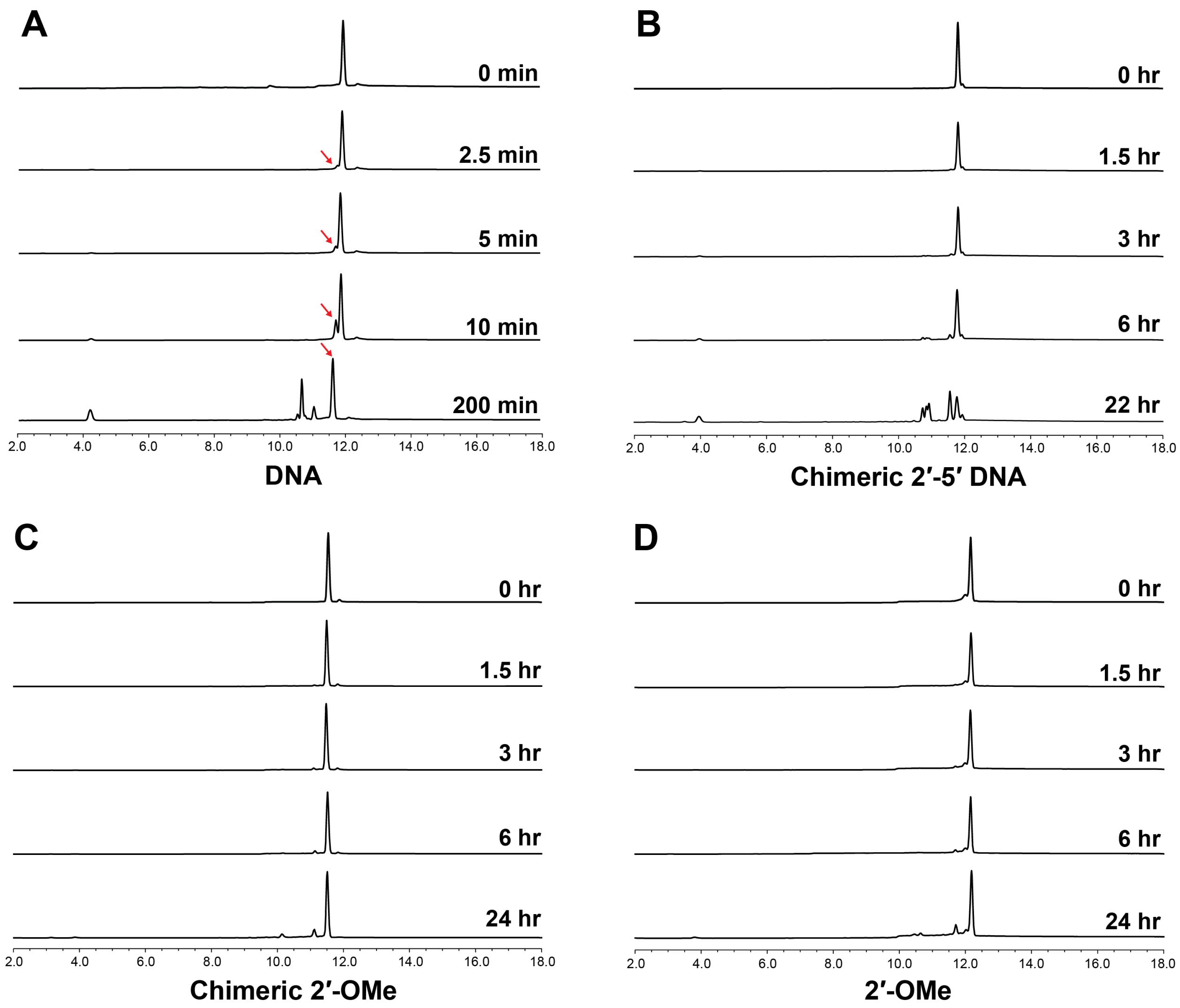

Supplementary Figure 3. Time-dependent HPLC analysis of acid-mediated cleavage of the asymmetric control strands. Degradation profiles observed for the asymmetric T_6AT_9 sequence as the fully DNA strand (A), chimeric DNA strand with a central 2'-5' linked adenosine (B), chimeric DNA strand with a central 2'-OMe adenosine (C), and fully 2'-OMe strand (D). Reactions were performed using 40 μM oligonucleotide in 120 mM citrate phosphate buffer (pH 3.3) at 90°C. Red arrow indicates the depurinated product, as supported by MALDI-TOF data (Supplementary Figure 4).

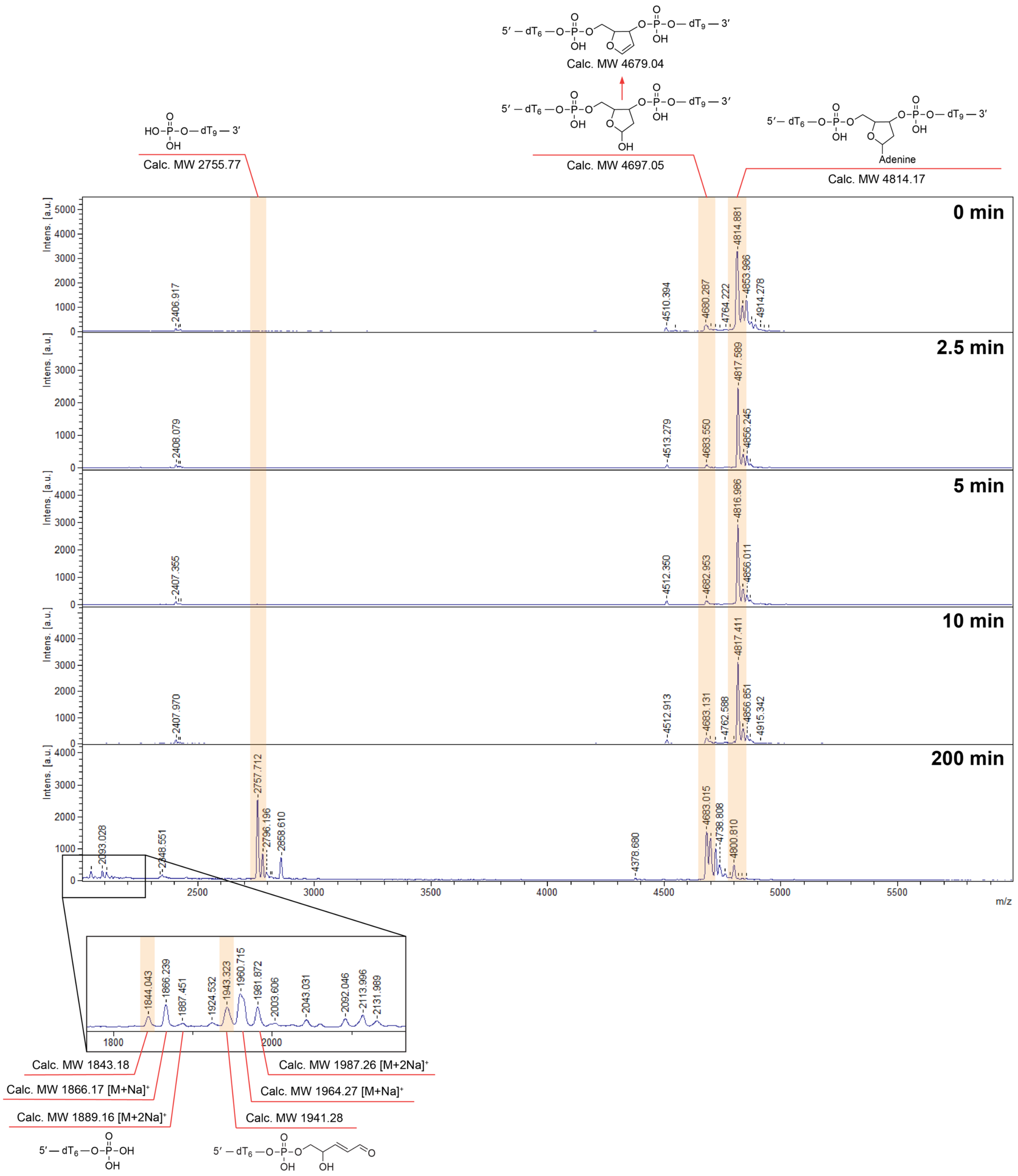

Supplementary Figure 4. Time-dependent mass spectrometry analysis of acid-mediated cleavage of DNA. The DNA oligonucleotide consisting of the asymmetric sequence 5'-T_6AT_9-3' (40 µM) was incubated in 120 mM citrate phosphate buffer (pH 3.3) at 90°C. Cleavage products (MW 1941.28 and MW 2755.77) from deprotonation of the alpha-proton during β-elimination were observed by MALDI-TOF. The depurinated form of the oligonucleotide (MW 4697.05) is observed as the dehydrated form (MW 4679.04). Some masses were observed with one or more bound sodium ions.

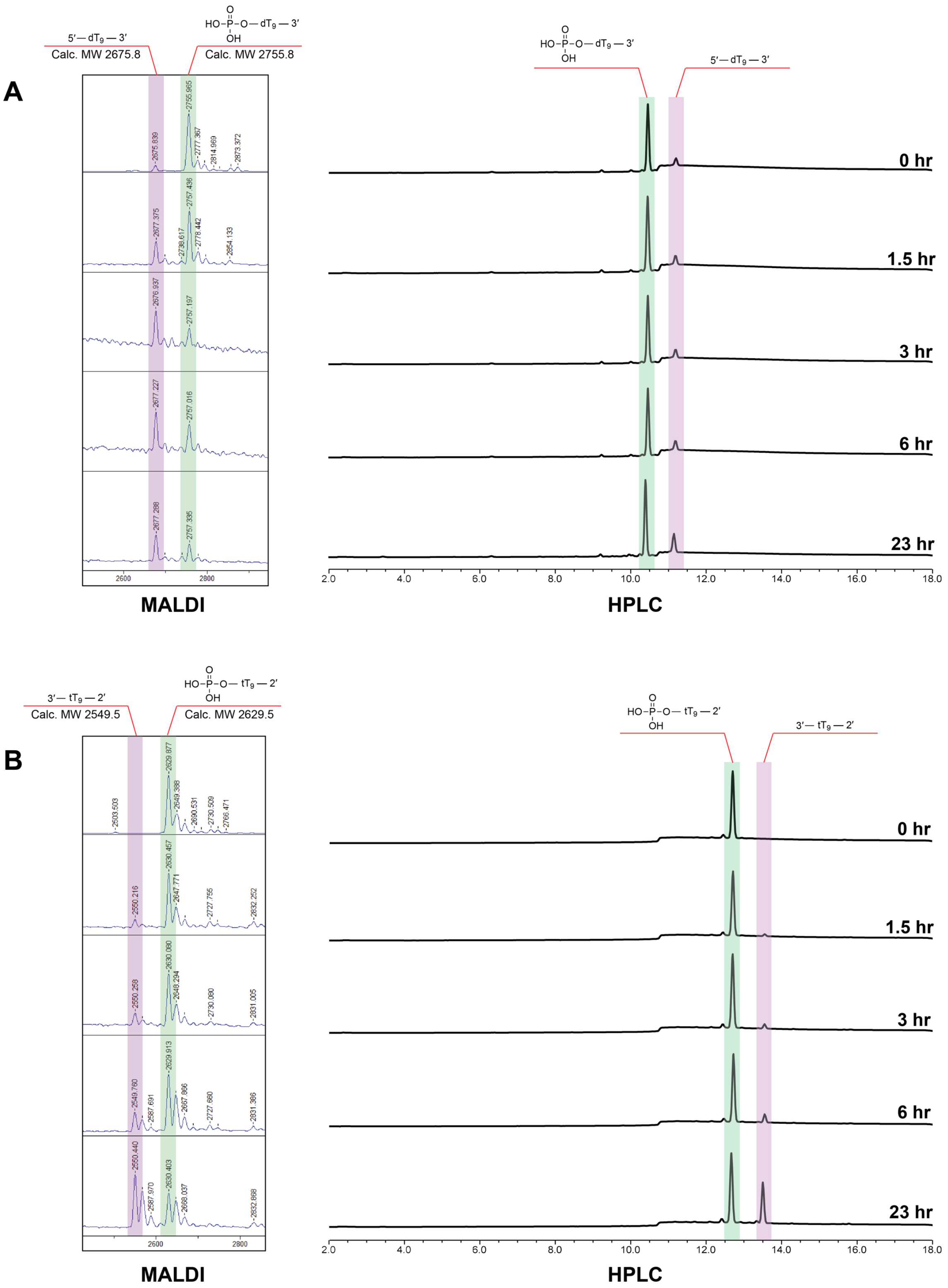

Supplementary Figure 5. Time-dependent HPLC and mass spectrometry analysis of acid hydrolysis of phosphorylated DNA and TNA. The oligonucleotides (40 µM) were incubated in 120 mM citrate phosphate buffer (pH 3.3) at 90°C. Mass spectra and HPLC chromatograms show the acid hydrolysis profile of A) 5'-phos-dT$_9$-3' and B) 3'-phos-tT$_9$-2'.

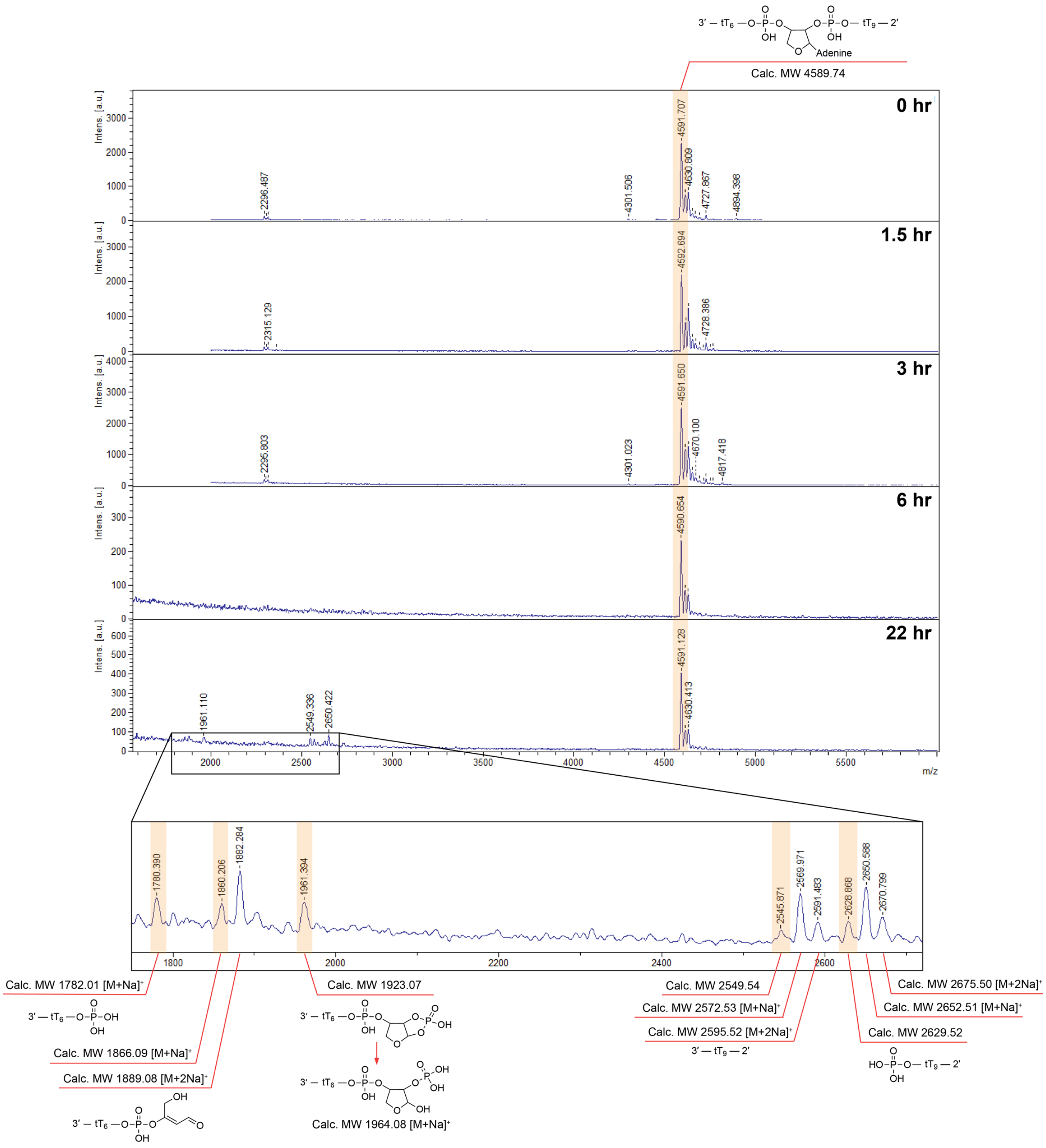

Supplementary Figure 6. Time-dependent mass spectrometry analysis of acid-mediated cleavage of TNA. The TNA oligonucleotide consisting of the asymmetric sequence 3'-T_6AT_9-2' (40 μM) was incubated in 120 mM citrate phosphate buffer (pH 3.3) at 90˚C. Cleavage products (MW 1866.09 and MW 2629.52) from deprotonation of the beta-proton during β-elimination were observed by MALDI-TOF. The observed mass of 1961.4 indicate a competing minor pathway (see Supplementary Figure 7). Some masses were observed with one or more bound sodium ions.

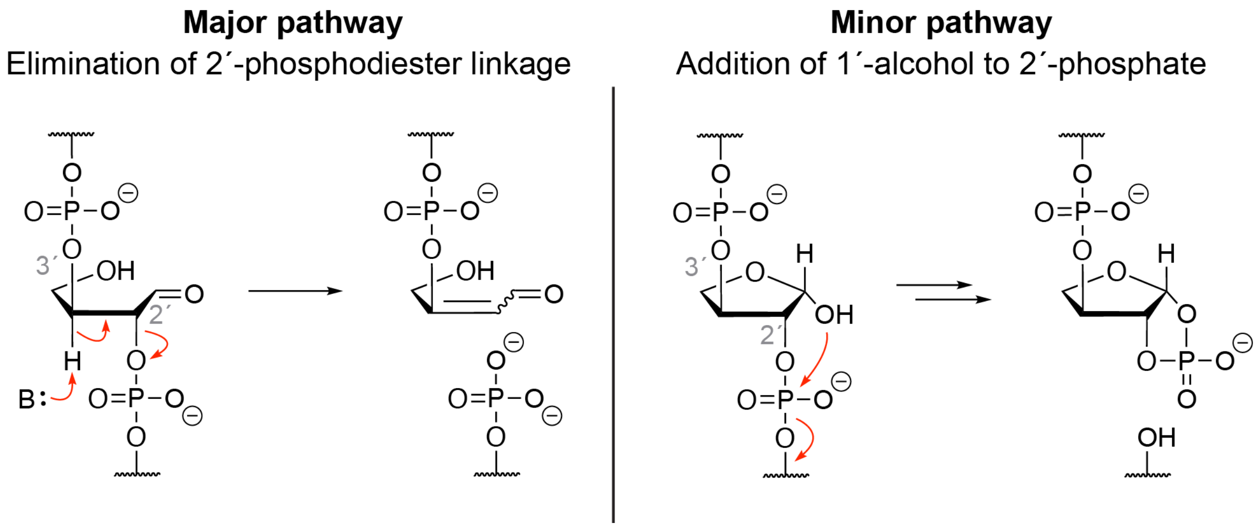

Supplementary Figure 7. Elimination pathways in acid-mediated TNA degradation. Based on mass spectrometry, TNA does not follow the same elimination pathway as DNA, where the proton alpha to the aldehyde is deprotonated to eliminate the 3'-phosphodiester linkage. Instead, the proton beta to the aldehyde is primarily deprotonated to eliminate the 2'-phosphodiester linkage in TNA. Our mass spectrometry data is also consistent with a competing minor pathway where the α-anomeric hydroxyl group adds to the 2'-phosphate, resulting in a cyclic phosphate and 3'-tT_9-2' without a 3´phosphate.

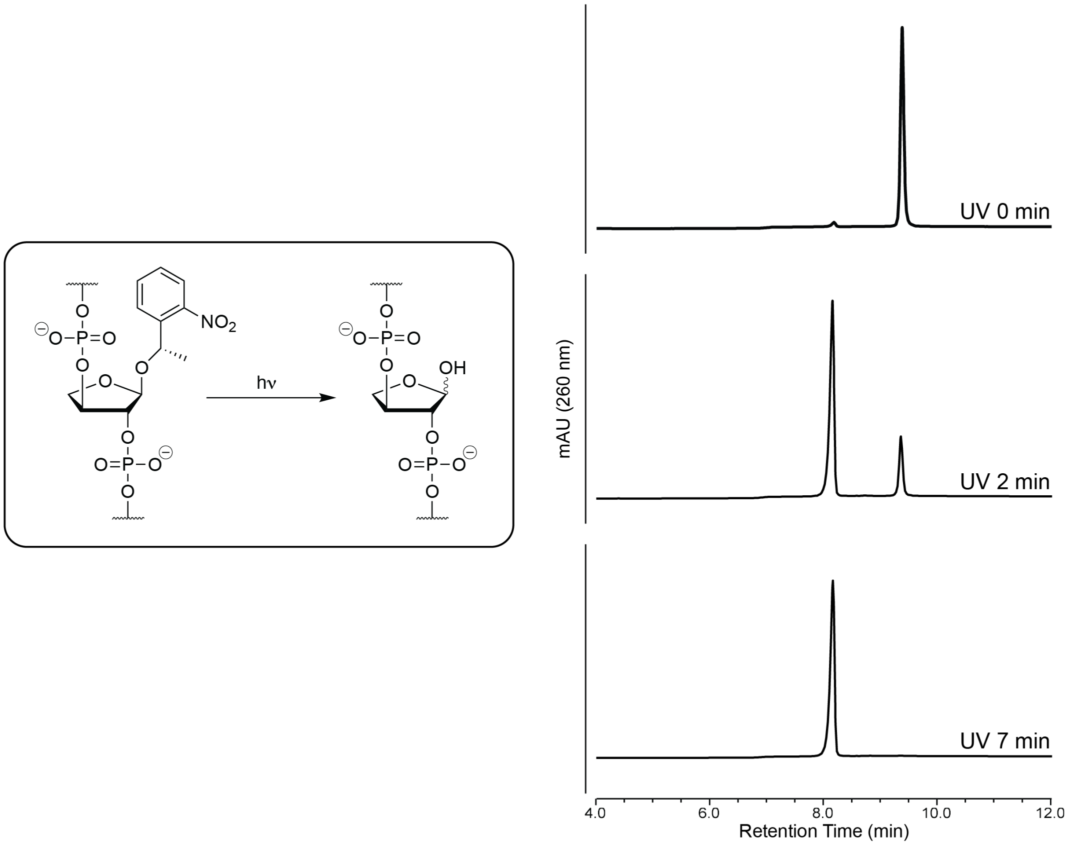

Supplementary Figure 8. HPLC analysis of NPE deprotection of asymmetric abasic TNA control strand via UV irradiation. NPE deprotection was monitored over different time intervals by RP-HPLC with an increasing gradient of 5% to 37% buffer B over 14 min with a flow rate of 1.00 mL/min at a set temperature of 40°C. The peak of the NPE-protected TNA (R_t = ~9.5 min) decreased while the peak of the abasic TNA (R_t = ~8.0 min) increased. Complete conversion was observed at 7 minutes of UV irradiation as described in the Materials and Methods.

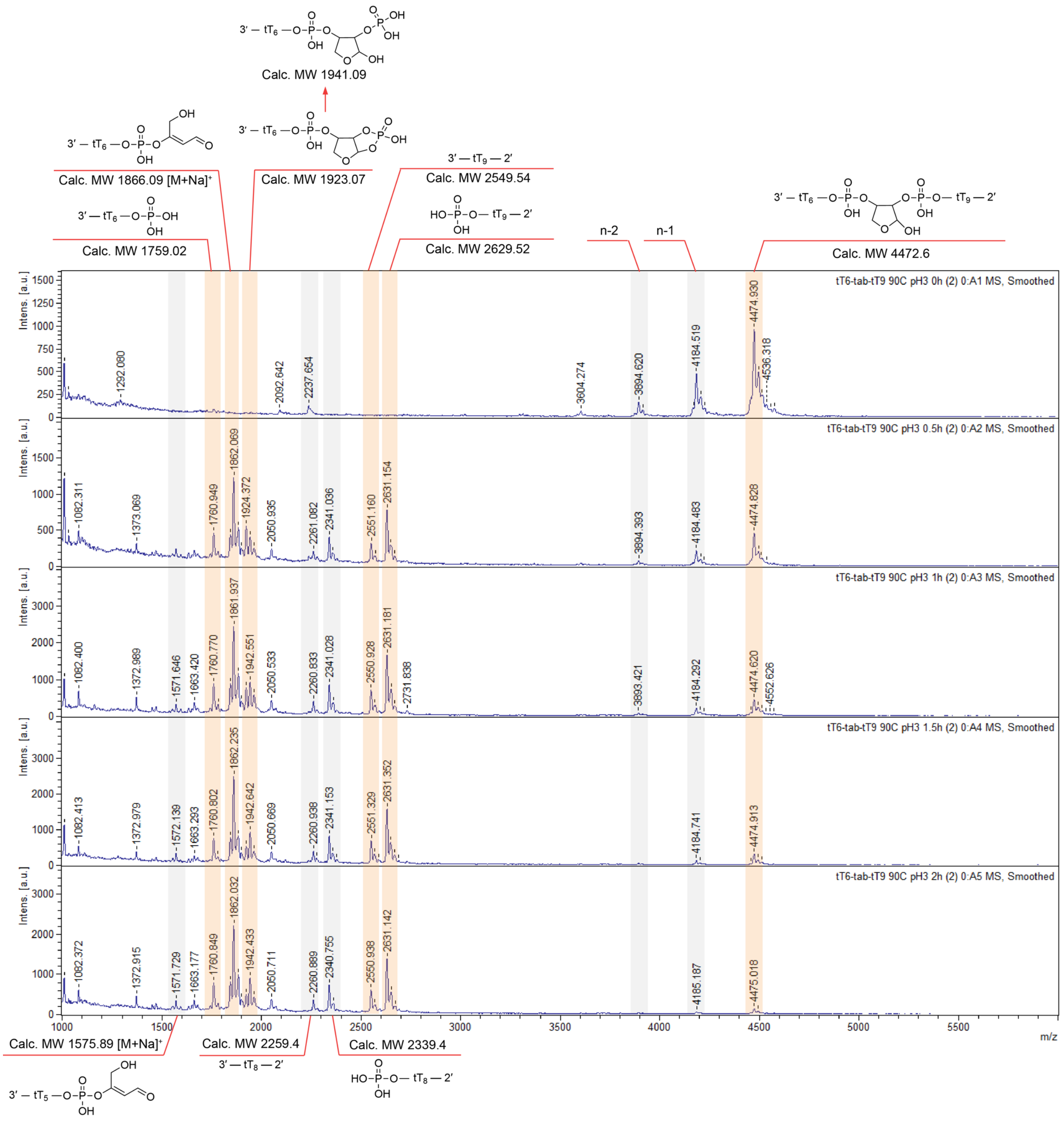

Supplementary Figure 9. Time-dependent mass spectrometry analysis of acid-mediated cleavage of TNA with an abasic residue. The TNA oligonucleotide consisting of the asymmetric sequence 3'-T_6-tAP-T_9-2' (tAP are abasic TNA residues) (40 µM) was incubated in 120 mM citrate phosphate buffer (pH 3.3) at 90˚C. Cleavage products (MW 1866.09 and MW 2629.52) from deprotonation of the beta-proton during β-elimination were observed by MALDI-TOF. The observed mass of 1942.4 indicate a competing minor pathway (see Supplementary Figure 7). Light gray bars highlight masses associated with n-1 and n-2 products and their cleavage products. Some masses were observed with one or more bound sodium ions.

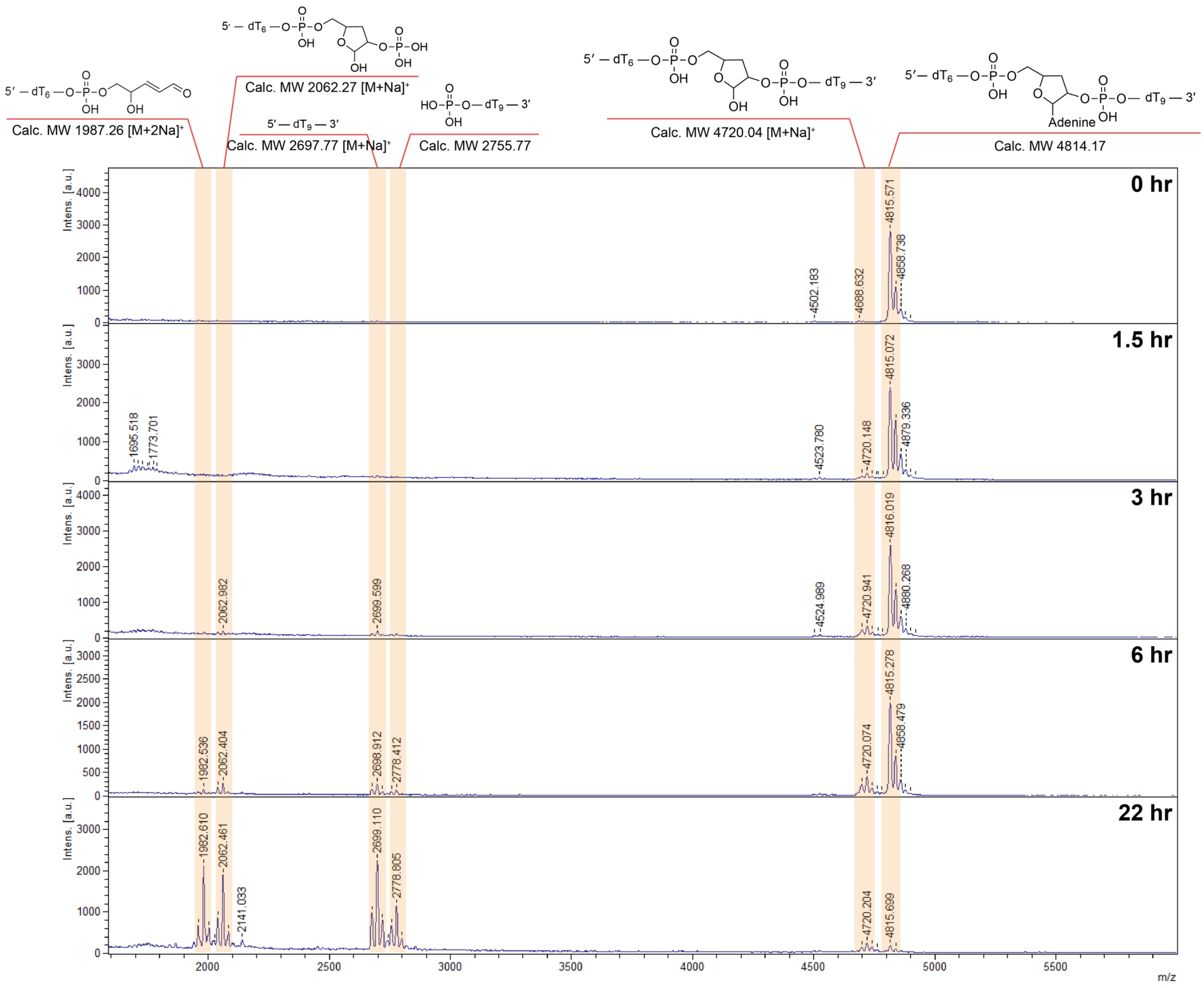

Supplementary Figure 10. Time-dependent mass spectrometry analysis of acid-mediated cleavage of a chimeric DNA with a central 2',5'-linked adenosine residue. The DNA oligonucleotide consisting of the asymmetric sequence 5'-$T_6\underline{A}T_9$-3' (underlined bases are 2',5'-linked DNA residues) (40 μM) was incubated in 120 mM citrate phosphate buffer (pH 3.3) at 90˚C. Cleavage products (MW 1987.26 and MW 2755.77) from deprotonation of the beta-proton during β-elimination were observed by MALDI-TOF. Like in the asymmetric TNA strands, the observed mass of 2062.5 indicate a similar competing mechanism involving a cyclic phosphate (see Supplementary Figure 7). Some masses were observed with one or more bound sodium ions.

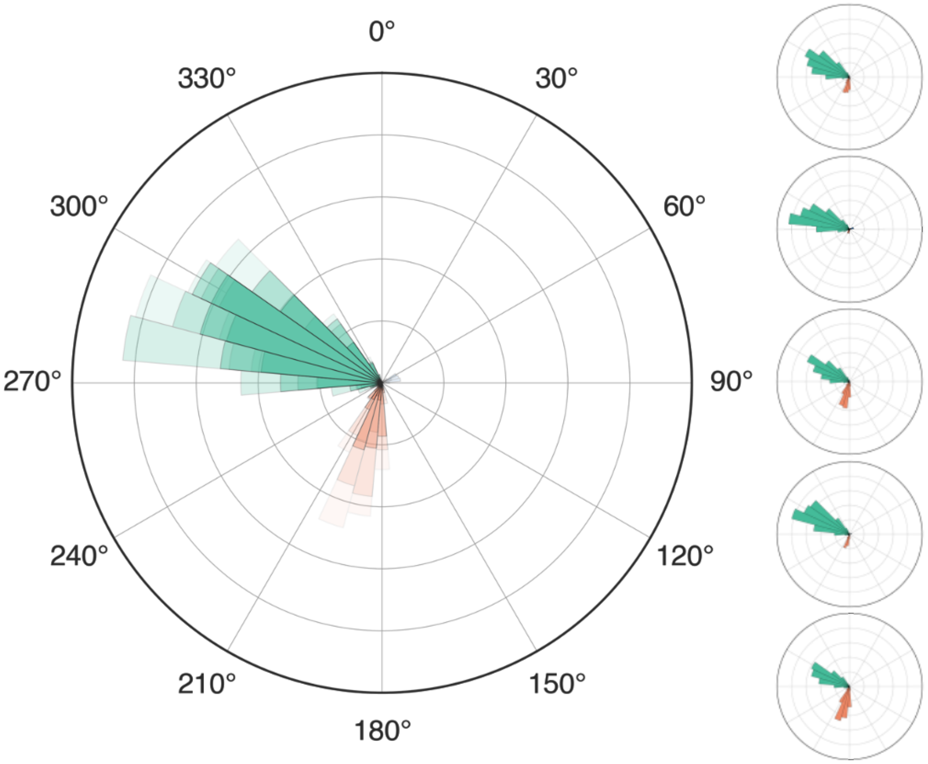

Supplementary Figure 11. Replication of conformational sampling. Data presented in Figure 6 was reproduced in five replicates of the molecular dynamics simulation. Results are shown overlaid for all replicates and individually. The average frequency of a conformation nearing the antiperiplanar conformation is 22.9 ± 2.7% (orange peaks).

Abasic TNA Phosphoramidite Synthesis.

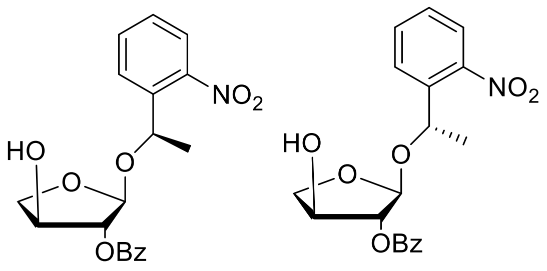

16a **16b**

(2S,3R,4S)-4-hydroxy-2-((S)-1-(2-nitrophenyl)ethoxy)tetrahydrofuran-3-yl benzoate (16a & 16b): To a solution of 1-(2-nitrophenyl)ethan-1-ol **14** (100 mg, 0.5982 mmol) and glycosyl donor **15** (362 mg, 0.7179 mmol) in dry CH_3CN, TMSOTf (37 μL, 0.2094 mmol) was added dropwise at -35°C under Argon atmosphere. The reaction mixture was stirred for 1 h (monitored by TLC) and quenched with 10 mL saturated aqueous $NaHCO_3$ solution. The aqueous layer was extracted with EtOAc (50 mL), and the organic layer was washed with brine, dried and concentrated under reduced pressure to give 200 mg inseparable mixture of diastereomers as crude product. To a solution of 200 mg of crude product in THF, 1M TBAF (0.36 mL, 0.3596 mmol) was added at 0°C. After stirring for 5 h at 0°C, the reaction mixture was concentrated, diluted with EtOAc (20mL). The organic layer was washed with water, dried and concentrated under reduced pressure to give crude. The crude was purified by column chromatography on silica gel (eluted with EtOAC:Hex = 1:4) to give separable diastereomers **16a** (60 mg, 0.1607 mmol, 27%) and **16b** (50 mg, 0.1339 mmol, 22%) in total 49% two-step yield.

16a: R_f 0.28 (EtOAc:Hex = 3:10); ^{1}H NMR (400 MHz, $CDCl_3$) δ 8.01-7.99 (m, 2H), 7.91 (d, *J* = 8.1 Hz, 1H), 7.82 (d, *J* = 7.9 Hz, 1H), 7.67-7.63 (m, 1H), 7.60-7.57 (m, 1H), 7.46-7.39 (m, 3H), 5.42 (q, *J* = 6.3 Hz, 1H), 5.38 (s, 1H), 5.18 (s, 1H), 4.32-4.29 (m, 1H), 4.10 (dd, *J* = 9.7, 6.3 Hz, 1H), 3.63 (dd, *J* = 9.7, 4.7 Hz, 1H), 3.11 (bs, 1H), 1.58 (d, *J* = 6.3 Hz, 3H); ^{13}C NMR (101 MHz, $CDCl_3$) δ 166.4, 147.3, 140.0, 133.7, 133.5, 129.9, 129.0, 128.6, 128.1, 128.0, 124.2, 105.3, 84.8, 75.6, 73.4, 71.3, 22.8; HRMS (ESI-TOF) calcd. for $C_{19}H_{19}NO_7Na$ $[M+Na]^+$ 396.1059, observed 396.1056.

16b: R_f 0.3 (EtOAc:Hex = 3:10); ^{1}H NMR (400 MHz, $CDCl_3$) δ 7.93-7.91 (m, 3H), 7.82-7.80 (d, *J* = 7.8 Hz, 1H), 7.68-7.65 (m, 1H), 7.57-7.53 (m, 1H), 7.44-7.38 (m, 3H), 5.52 (q, *J* = 6.2 Hz, 1H), 5.18 (s, 1H), 4.96 (s, 1H), 4.35 (s, 1H), 4.36-4.31 (m, 1H), 4.08-4.03 (m, 1H), 3.21 (bs, 1H), 1.59 (d, *J* = 6.3 Hz, 3H); ^{13}C NMR (101 MHz, $CDCl_3$) δ 166.2, 148.5, 138.4, 133.8, 133.6, 129.8, 129.0, 128.5, 128.4, 128.0, 124.5, 103.5, 84.1, 75.4, 73.9, 69.6, 24.1; HRMS (ESI-TOF) calcd. for $C_{19}H_{19}NO_7Na$ $[M+Na]^+$ 396.1059, observed 396.1042.

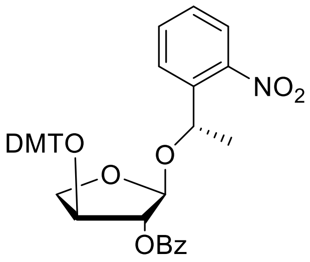

(2S,3R,4S)-4-(bis(4-methoxyphenyl)(phenyl)methoxy)-2-((S)-1-(2-nitrophenyl)ethoxy) tetrahydrofuran-3-yl benzoate (17): To a solution of **16b** (40 mg, 0.1071 mmol) in CH_2Cl_2 (1.5 mL), collidine (42 µL, 0.3214 mmol) and DMTCl (54 mg, 0.1607 mmol) were added followed by the addition of $AgNO_3$ (9 mg, 0.0535 mmol). The reaction mixture was stirred at 37°C for 5 h, quenched with water and diluted with CH_2Cl_2. The organic layer was separated, dried and concentrated under reduced pressure to give crude. The crude was purified by column chromatography (on TEA-deactivated silica, eluted with 1.5:10 EtOAc:Hex) to give product **17** (40 mg, 0.0592 mmol, 55%) as colorless oil. R_f 0.7 (EtOAc:Hex = 3:10); ^{1}H NMR (400 MHz, $CDCl_3$) δ 7.98 (d, *J* = 7.8 Hz, 1H), 7.90-7.85 (m, 3H), 7.63 (t, *J* = 6.3 Hz, 1 H), 7.55-7.48 (m, 3H), 7.40-7.33 (m, 7H), 7.27-7.23 (m, 2H), 7.20-7.16 (m, 1H), 6.78-6.73 (m, 4H), 5.43 (q, *J* = 6.3 Hz, 1H), 5.12 (d, *J* = 1.4 Hz, 1H), 4.59 (s, 1H), 4.38-4.35 (m, 1H), 3.73 (dd, *J* = 9.1, 7.0 Hz, 1H), 3.69 (s, 3H), 3.66 (s, 3H), 3.55 (dd, *J* = 9.1, 7.0 Hz, 1H), 1.62 (d, *J* = 6.3 Hz, 3H); ^{13}C NMR (101 MHz, $CDCl_3$) δ 165.3, 158.7, 158.7, 148.3, 145.2, 139.1, 136.2, 136.1, 133.9, 133.2, 130.3, 130.2, 129.8, 129.4, 128.5, 128.3, 128.1, 128.0, 128.0, 127.0, 124.3, 113.4, 113.3, 103.4, 87.2, 83.8, 77.3, 71.1, 68.7, 55.1, 55.1, 24.1; HRMS (ESI-TOF) calcd. for $C_{40}H_{37}NO_9Na$ $[M+Na]^+$ 698.2366, observed 698.2372.

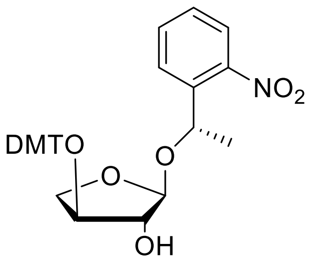

(2S,3R,4S)-4-(bis(4-methoxyphenyl)(phenyl)methoxy)-2-((S)-1-(2-nitrophenyl)ethoxy) tetrahydrofuran-3-ol (18): A solution of **17** (40 mg, 0.0592 mmol) in 3 mL of THF:MeOH:H_2O (0.012 M, 3.5:3:1) was cooled to 0°C and 0.90 mL (0.9058 mmol) of 1M NaOH was added to it. After stirring for 5 h at 0°C, 54 mg (1.0063 mmol) of solid NH_4Cl was added and stirring was continued for 20 min. The reaction mixture was evaporated to remove solvents and the crude was purified by column chromatography (on TEA-deactivated silica, eluted with 2:10 EtOAc:Hex) to give product **18** (25 mg, 0.0437 mmol, 73% yield) as colorless oil. R_f 0.4 (EtOAc:Hex = 3:10); ^{1}H NMR (400 MHz, $CDCl_3$) 7.86 (d, *J* = 8.1 Hz, 1H), 7.75 (d, *J* = 7.9 Hz, 1H), 7.61-7.59 (m, 1 H), 7.50-7.48 (m, 2H), 7.40-7.36 (m, 5H), 7.32-7.29 (m, 2H), 7.25-7.23 (m, 1H), 6.86-6.83 (m, 4H), 5.33 (q, *J* = 6.3 Hz, 1H), 4.50 (s, 1H), 4.00-3.97 (m, 1H), 3.88 (m, 1H), 3.79 (s, 6H), 3.69-3.60 (m, 2H), 1.59 (d, *J* = 6.3 Hz, 3H); ^{13}C NMR (101 MHz, $CDCl_3$) δ ; 158.8, 148.5, 145.4, 139.1, 136.7, 136.6, 133.5, 130.2, 128.3, 128.2, 128.1, 128.0, 127.1, 124.3, 113.3, 105.5, 86.7, 82.2, 80.0, 71.5, 69.1, 55.3, 24.0; HRMS (ESI-TOF) calcd. for $C_{33}H_{33}NO_8Na$ $[M+Na]^+$ 594.2104, observed 594.2093.

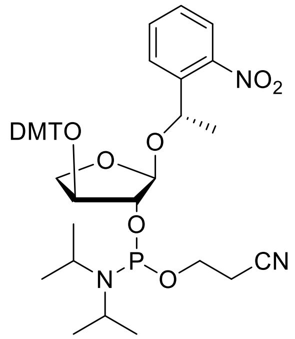

(2S,3R,4S)-4-(bis(4-methoxyphenyl)(phenyl)methoxy)-2-((S)-1-(2-nitrophenyl)ethoxy) tetrahydrofuran-3-yl (2-cyanoethyl) diisopropylphosphoramidite (19): To a suspension of **18** (60 mg, 0.1049 mmol) and DMAP (2.5 mg, 0.0210 mmol) in CH_2Cl_2 (10 mL) was added *N*,*N*-diisopropylethylamine (DIPEA) (27 µL, 0.1574 mmol), followed by the addition of 2-cyanoethyl-*N*,*N*-diisopropylchlorophosphoramidite (35 µL, 0.1574 mmol). After stirring for 40 minutes at room temperature, the solution was diluted with CH_2Cl_2 (20 mL) and washed with saturated aqueous $NaHCO_3$ (40 mL). The organic layer was washed with brine, dried and concentrated under reduced pressure to give crude. The crude was purified by column chromatography on silica gel (eluted with 1.5:10 EtOAc:Hex) to afford product **19** (77 mg, 0.1010 mmol, 96%) as white solid. TLC (EtOAc:Hex, 3:10). R_f 0.8 (EtOAc:Hex = 3:10); ^{31}P NMR (162 MHz, CD3CN) δ 149.3, 149.0; HRMS (ESI-TOF) calcd. for $C_{42}H_{50}N_3O_9PNa$ $[M+Na]^+$ 794.3182, observed 794.3160.

Compound Characterization.

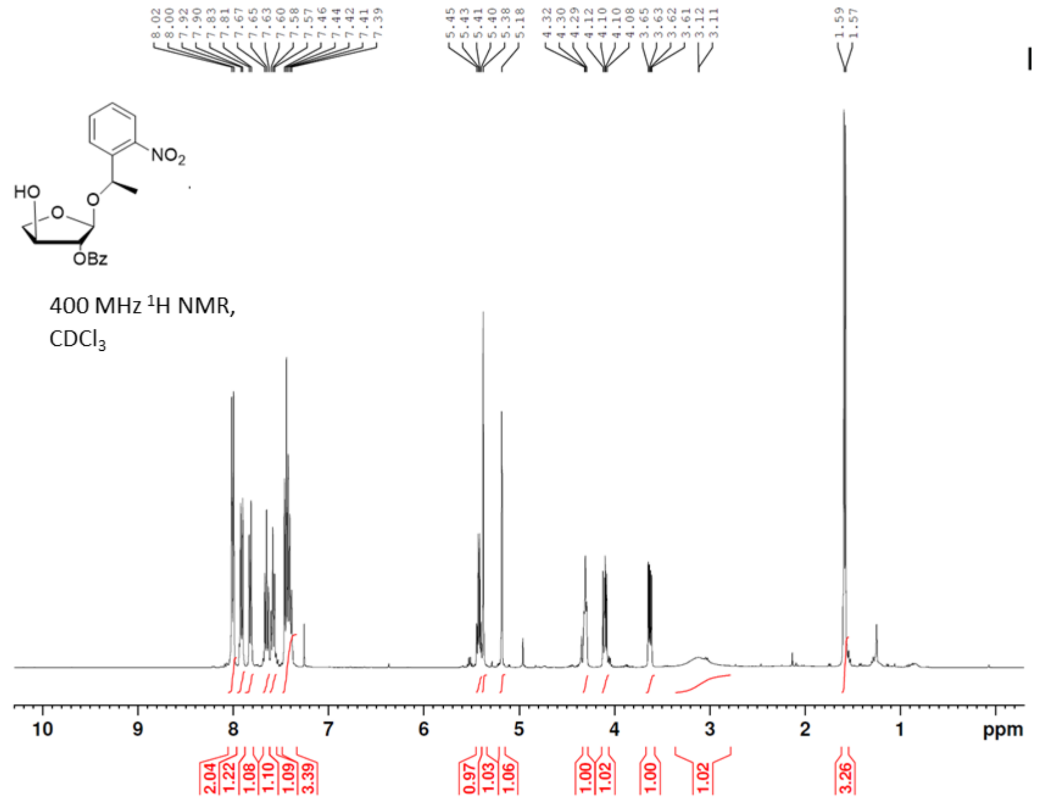

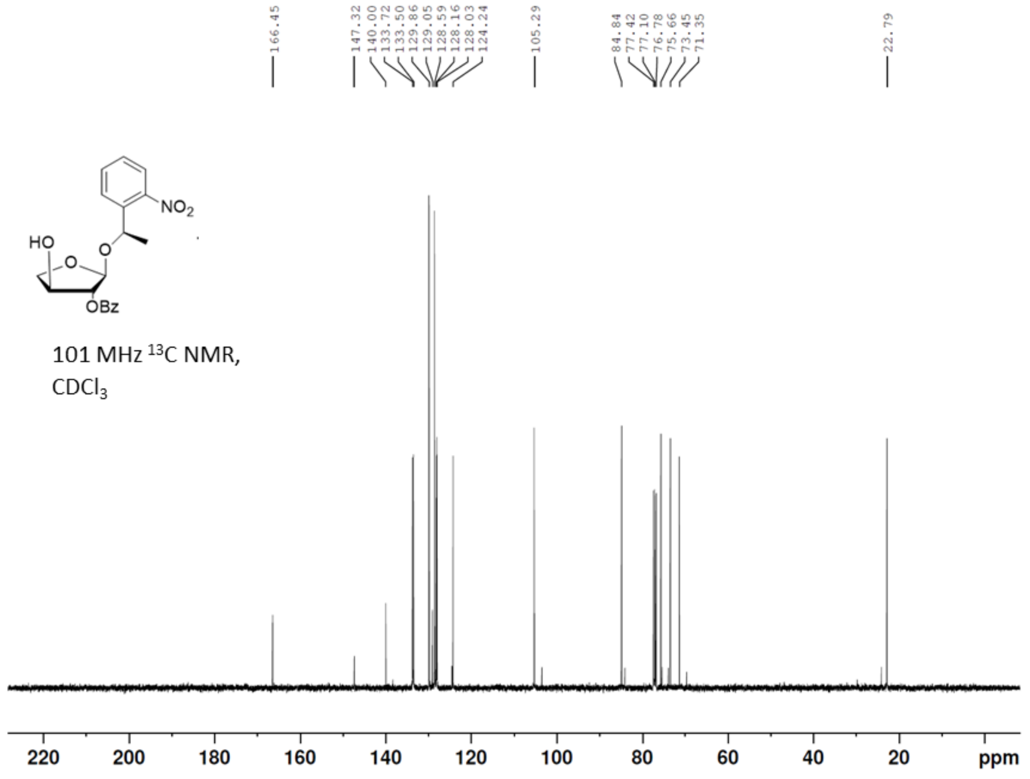

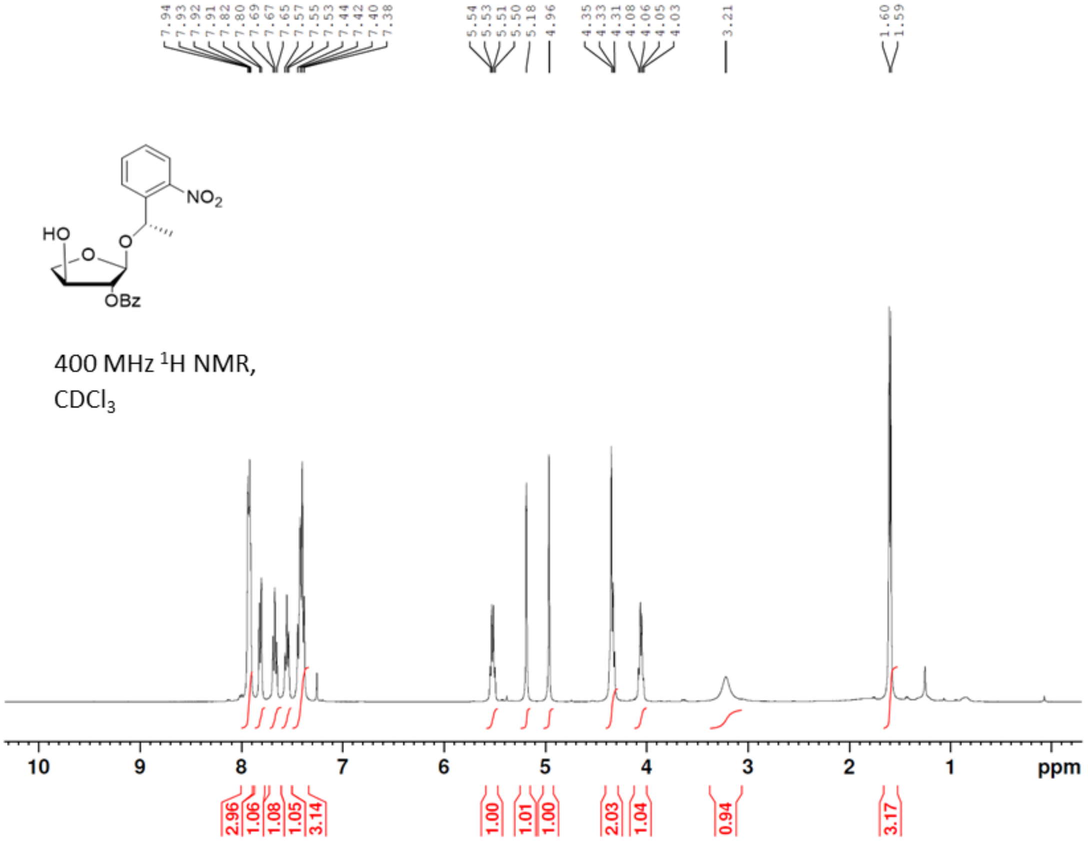
7.94 7.93 7.92 7.91 7.82 7.80 7.69 7.67 7.65 7.57 7.55 7.53 7.44 7.42 7.40 7.38
5.54 5.53 5.51 5.50 5.18 4.96
4.35 4.33 4.31 4.08 4.06 4.05 4.03
3.21
1.60 1.59
NO_2
HO
O
O
OBz
400 MHz ^{1}H NMR,
$CDCl_3$
2.96 1.06 1.08 1.05 3.14 1.00 1.01 1.00 2.03 1.04 0.94 3.17
10 9 8 7 6 5 4 3 2 1 ppm

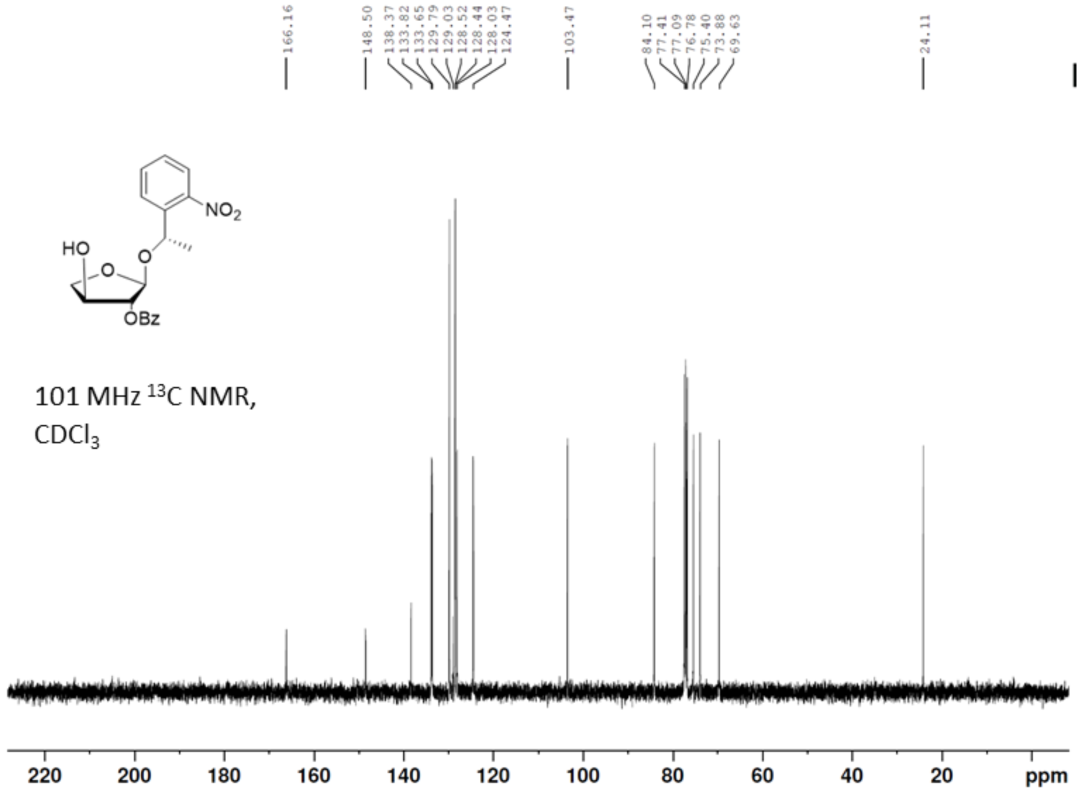
166.16
148.50 138.37 133.82 133.65 129.79 129.03 128.52 128.44 128.03 124.47
103.47
84.10 77.41 77.09 76.78 75.40 73.88 69.63
24.11
NO_2
HO
O
O
OBz
101 MHz ^{13}C NMR,
$CDCl_3$
220 200 180 160 140 120 100 80 60 40 20 ppm

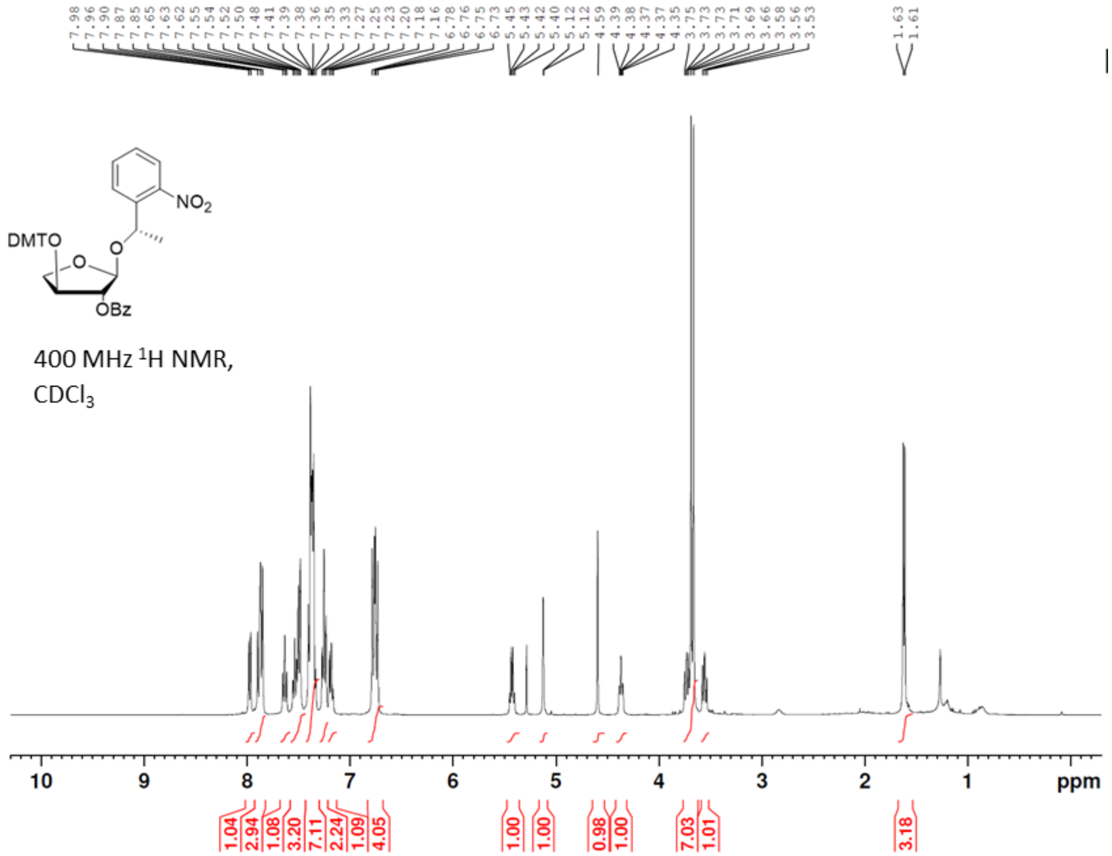
7.98 7.96 7.90 7.87 7.85 7.65 7.63 7.62 7.55 7.54 7.52 7.50 7.48 7.41 7.39 7.38 7.36 7.35 7.33 7.27 7.25 7.23 7.20 7.18 7.16 6.78 6.76 6.75 6.73 5.45 5.43 5.42 5.40 5.12 5.12 4.59 4.39 4.38 4.37 4.37 4.35 3.75 3.73 3.73 3.71 3.69 3.66 3.58 3.56 3.53 1.63 1.61
NO2
DMTO
O
O
OBz
400 MHz 1H NMR,
CDCl3
10 9 8 7 6 5 4 3 2 1 ppm
1.04 2.94 1.08 3.20 7.11 2.24 1.09 4.05 1.00 1.00 0.98 1.00 7.03 1.01 3.18

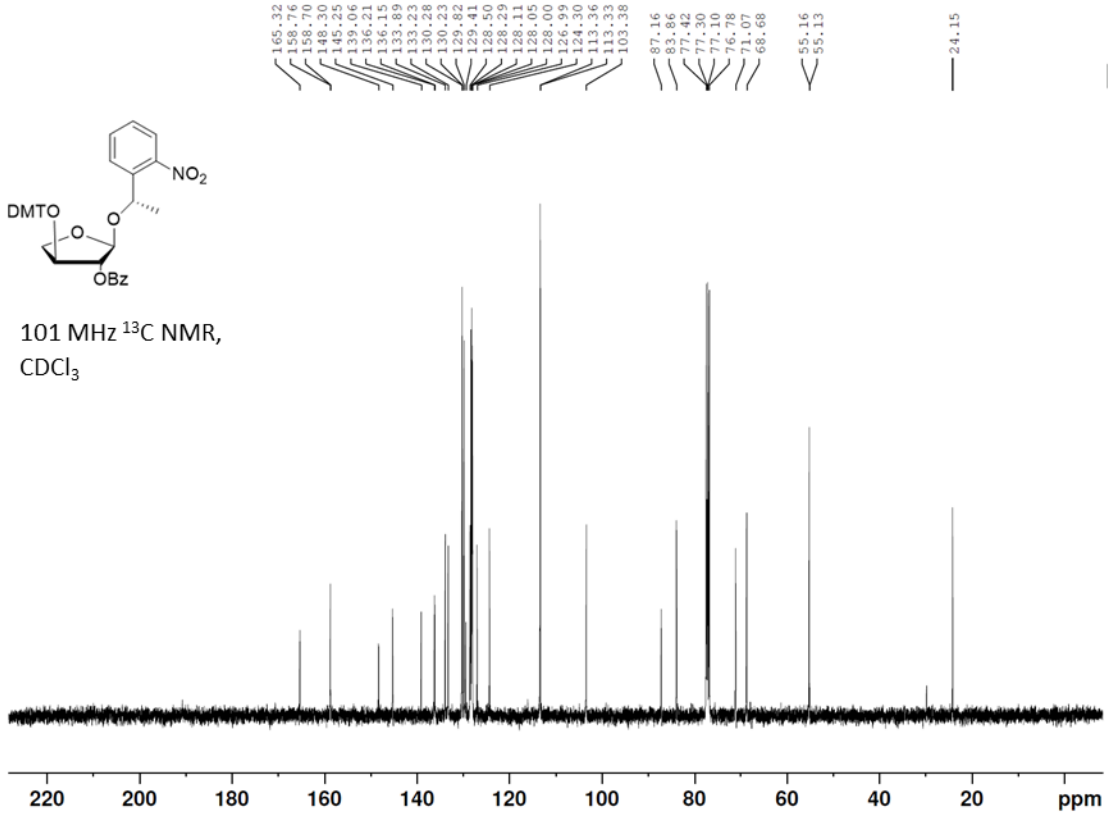
165.32 158.76 158.70 148.30 145.25 139.06 136.21 136.15 133.89 133.23 130.28 130.23 129.82 129.41 128.50 128.29 128.11 128.05 128.00 126.99 124.30 113.36 113.33 103.38 87.16 83.86 77.42 77.30 77.10 76.78 71.07 68.68 55.16 55.13 24.15
NO2
DMTO
O
O
OBz
101 MHz 13C NMR,
CDCl3
220 200 180 160 140 120 100 80 60 40 20 ppm

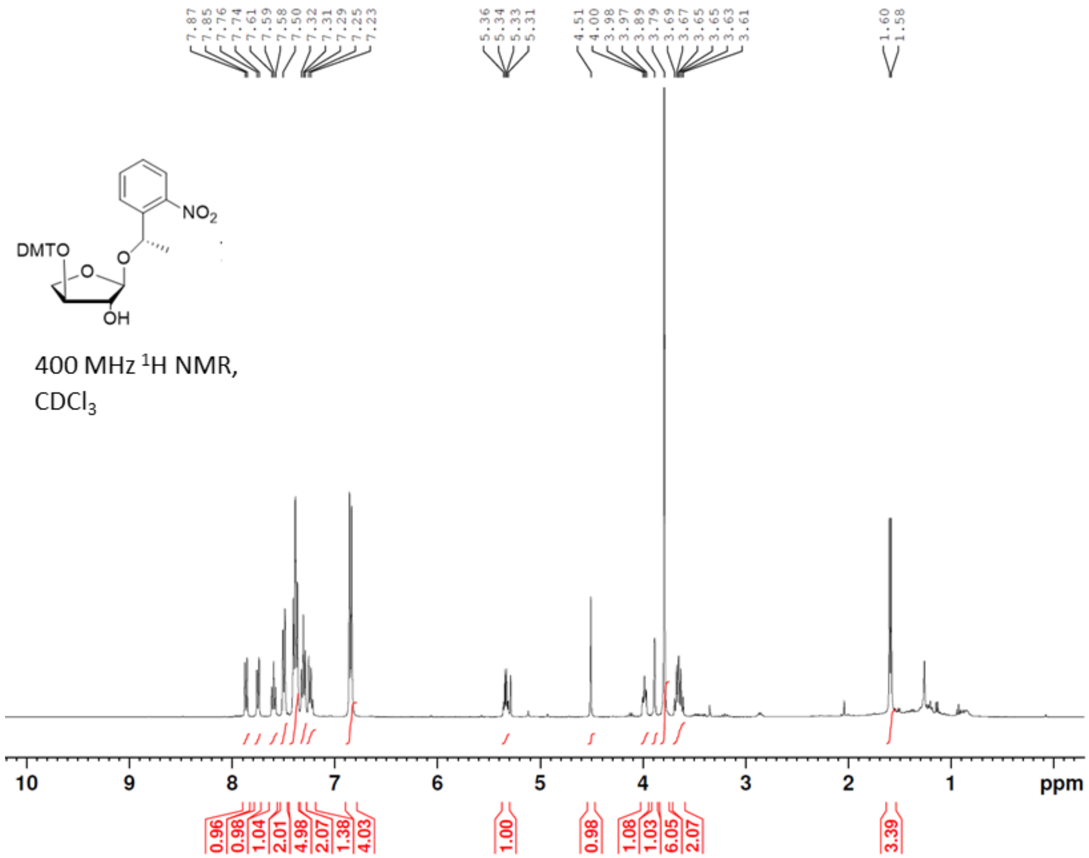

7.87 7.85 7.76 7.74 7.61 7.59 7.58 7.50 7.32 7.31 7.29 7.25 7.23
5.36 5.34 5.33 5.31
4.51 4.00 3.98 3.97 3.89 3.79 3.69 3.67 3.65 3.63 3.61
1.60 1.58
NO2
DMTO
O
O
OH
400 MHz 1H NMR, CDCl3
0.96 0.98 1.04 2.01 4.98 2.07 1.38 4.03
1.00
0.98
1.08 1.03 6.05 2.07
3.39
10 9 8 7 6 5 4 3 2 1 ppm

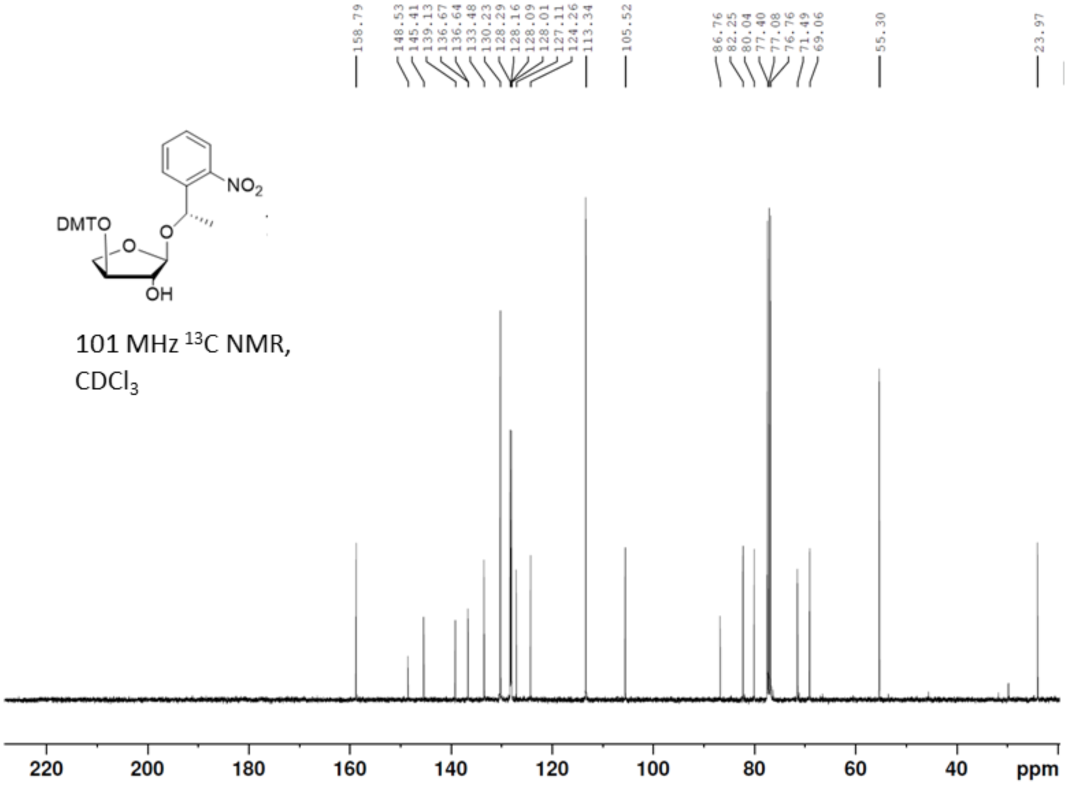

158.79
148.53 145.41 139.13 136.67 136.64 133.48 130.23 128.29 128.16 128.09 128.01 127.11 124.26 113.34
105.52
86.76 82.25 80.04 77.40 77.08 76.76 71.49 69.06
55.30
23.97
NO2
DMTO
O
O
OH
101 MHz 13C NMR, CDCl3
220 200 180 160 140 120 100 80 60 40 ppm

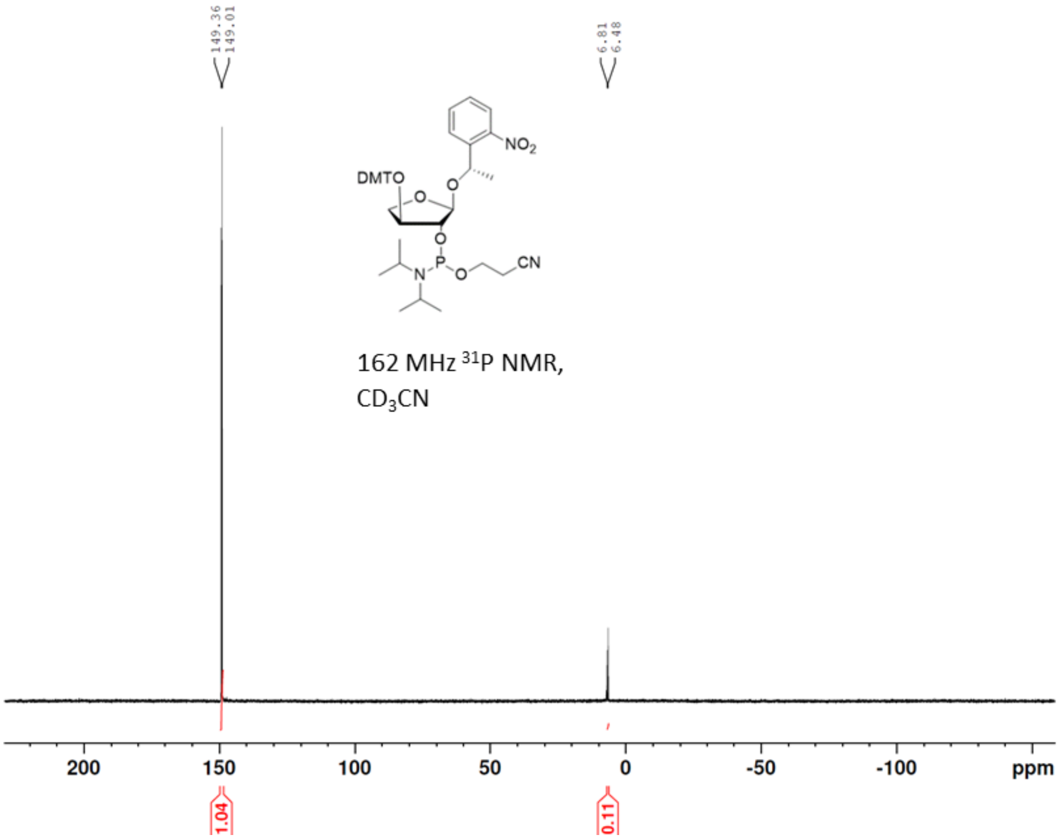
149.36
149.01
6.81
6.48
DMTO
NO2
CN
162 MHz 31P NMR,
CD3CN
200
150
100
50
0
-50
-100
ppm
1.04
0.11
